# Supplementary material for: Correlations as a resource in quantum thermodynamics
Source: Nat Commun. 2019 Jun 7;10:2492. doi: 10.1038/s41467-019-10572-8 (PMC6555829; doi:10.1038/s41467-019-10572-8)
Supplement: Supplementary file 1 — Supplementary Information [file 41467_2019_10572_MOESM1_ESM.pdf]

Supplementary Information – Correlations as a resource in quantum thermodynamics

F. Sapienza, F. Cerisola, A. J. Roncaglia

Supplementary Note 1. c-work of formation

In this work we focus our study on the process of formation of N correlated copies of a system S with non-trivial Hamiltonian H_{S}. More specifically, given a state ρ of the system with $[\rho, H_{\mathrm{S}}]=0$ and a state $\rho^{(N)}$ which satisfies

$$\operatorname{tr}_{-i}\left(\rho^{(N)}\right)=\rho \quad \forall i=1,2,\dots,N, \tag{1}$$

where $\operatorname{tr}_{-i}(\cdot)=\operatorname{tr}_{1,2,\dots,i-1,i+1,\dots,N}(\cdot)$ is the partial trace over all subsystems except for the i-th one, we consider the transformation via thermal operations given by $\tau_{\mathrm{S}}^{\otimes N}\otimes |W\rangle\langle W| \to \rho^{(N)}\otimes|0\rangle\langle 0|$, where τ_{S} is the Gibbs state of S. The aim is to find states $\rho^{(N)}$ with minimum work of formation W. We can formalize this problem as a minimization problem over a set of feasible solutions.

Definition 1 (feasible states). Given a state ρ of the system S and $N\in\mathbb{N}$, let $\mathcal{C}(\rho,N)$ be the set of states of the N copies, $\mathrm{S}^{\otimes N}$, given by

$$\mathcal{C}(\rho,N)=\left\{\rho^{(N)}:\operatorname{tr}_{-i}\left(\rho^{(N)}\right)=\rho \quad \forall i=1,2,\dots,N\right\}. \tag{2}$$

Except for the product state $\rho^{\otimes N}$, the set $\mathcal{C}(\rho,N)$ is formed by correlated states of the N subsystems. The c-work of formation is then defined as the minimum amount of energy that is required to create a state in $\mathcal{C}(\rho,N)$ from the Gibbs state of the total system.

Definition 2 (work of formation of correlated copies). Given a state ρ of the system S and a number of copies N, we define the work of formation of correlated copies or just c-work of formation, denoted as $\mathcal{W}_{\mathrm{form}}(\rho,N)$, by

$$\mathcal{W}_{\mathrm{form}}(\rho,N)=\min_{\rho^{(N)}\in\mathcal{C}(\rho,N)} W_{\mathrm{form}}\left(\rho^{(N)}\right). \tag{3}$$

Let D be the dimension of the system S and $E_1\le E_2\le\ldots\le E_D$ the eigenvalues of H_{S}. Let's assume that there are at least two different eigenvalues (a similar treatment can be considered for trivial Hamiltonians and the solution is similar to the one that is obtained in the limit $\beta\to 0$). Then the states ρ and $\rho^{(N)}$ can be written as

$$\rho=\sum_{d=1}^{D} p_d\,|E_d\rangle\langle E_d|, \tag{4}$$

$$\rho^{(N)}=\sum_{(d_1,\dots,d_N)\in\{1,\dots,D\}^N}\lambda_{d_1,d_2,\dots,d_N}\,|E_{d_1},E_{d_2},\dots,E_{d_N}\rangle\langle E_{d_1},E_{d_2},\dots,E_{d_N}|, \tag{5}$$

where p_d are the eigenvalues of the state ρ and $\lambda_{d_1,d_2,\dots,d_N}$ are the occupation probabilities of the state $\rho^{(N)}$ with the i-th subsystem in the energy state E_{d_i}. Applying $\operatorname{tr}_{-j}(\cdot)$ in Supplementary Equation (5) we find that the constraint of Supplementary Equation (1) on the partial traces will be satisfied if and only if

$$p_d=\sum_{(d_1,\dots,d_{j-1},d_{j+1},\dots,d_N)\in\{1,\dots,D\}^{N-1}}\lambda_{d_1,\dots,d_{j-1},d,d_{j+1},\dots,d_N} \quad \forall\, d,j. \tag{6}$$

On the other hand, the Gibbs state of the N copies is given by

$$\tau_{\mathrm{S}}^{\otimes N}=\sum_{(d_1,\dots,d_N)\in\{1,\dots,D\}^N}\frac{e^{-\beta\left(E_{d_1}+E_{d_2}+\ldots+E_{d_N}\right)}}{Z_{\mathrm{S}}^N}\,|E_{d_1},E_{d_2},\dots,E_{d_N}\rangle\langle E_{d_1},E_{d_2},\dots,E_{d_N}|, \tag{7}$$

where $Z_{\mathrm{S}}=\sum_{i=1}^{D}e^{-\beta E_i}$ is the partition function of the system S at inverse temperature $\beta=(k_{\mathrm{B}}T)^{-1}$. Then, the work of formation of $\rho^{(N)}$ is

$$W_{\mathrm{form}}(\rho^{(N)})=k_{\mathrm{B}}T\log\max_{d_1,\dots,d_N}\left\{\lambda_{d_1,\dots,d_N}e^{\beta\left(E_{d_1}+\ldots+E_{d_N}\right)}Z_{\mathrm{S}}^N\right\}. \tag{8}$$

Let p_E be the occupation of energy level E of the N-partite system, which is given by

$$p_E=\sum_{d_1,\dots,d_N\,:\,\sum_{i=1}^N E_{d_i}=E}\lambda_{d_1,d_2,\dots,d_N}, \tag{9}$$

and let $g_N(E)$ be its degeneracy (i.e. the number of terms in the sum of Supplementary Equation (9)). Then, it is clear from Supplementary Equation (8) that in order to minimize the work of formation, the distribution of p_E in the $g_N(E)$ states should be uniform:

$$\lambda_{d_1,\ldots,d_N} = \frac{p_{\sum_{i=1}^N E_{d_i}}}{g_N\left(\sum_{i=1}^N E_{d_i}\right)} \equiv \lambda_{\sum_{i=1}^N E_{d_i}}. \tag{10}$$

Given this symmetry, we introduce the notation $\lambda_E = \lambda_{d_1,\ldots,d_N}$, with $E = \sum_{i=1}^N E_{d_i}$. Then, we can restrict the search for a state $\rho^{(N)}$ that minimizes $\mathcal{W}_{\text{form}}$ to states of the following form

$$\rho^{(N)} = \sum_E p_E \sum_{\psi\,:\,E(\psi)=E} \frac{1}{g_N(E)} |\psi\rangle\langle\psi| . \tag{11}$$

This simplifies the problem of finding the state $\rho^{(N)}$ that minimizes the work of formation. The state of Supplementary Equation (11) is completely characterized by its energy distribution among the set $\mathcal{E}_N$ of energies of the N-partite system. Now the constraints of Supplementary Equation (6) on the reduced states take a simpler form

$$\begin{aligned} p_d &= \sum_{(d_1,\ldots,d_{j-1},d_{j+1},\ldots,d_N)\in\{1,\ldots,D\}^{N-1}} \lambda_{d_1,\ldots,d_{j-1},d,d_{j+1},\ldots,d_N} \\ &= \sum_{E\in\mathcal{E}_N} g_{N-1}(E-E_d)\lambda_E \quad \forall d = 1,2,\ldots,D, \end{aligned} \tag{12}$$

where λ_E is any of the eigenvalues of $\rho^{(N)}$ with energy E. Notice that each of the terms $g_{N-1}(E-E_d)\lambda_E$ represents the conditional probability that one of the copies have local energy E_d given that the total energy is E. The following proposition summarizes the previous analysis.

Proposition 1. Given a local state ρ and $N \in \mathbb{N}$, it holds

$$\min_{\rho^{(N)}\in\mathcal{C}(\rho,N)} W_{\text{form}}(\rho^{(N)}) = \min_{\rho^{(N)}\in\mathcal{C}^*(\rho,N)} W_{\text{form}}(\rho^{(N)}), \tag{13}$$

where $\mathcal{C}^*(\rho,N)$ is the set of states of the form given by Supplementary Equation (11) which satisfies Supplementary Equation (12).

A. c-work of formation as a linear program

From the results of the previous section it is clear that the search for the state $\rho^{(N)}$ with minimum work of formation is equivalent to solving the following constrained minimization problem

$$\begin{aligned} \min_{\{\lambda_E\}_{E\in\mathcal{E}_N}} \quad & k_{\mathrm{B}}T \log\left[\max_E \left\{\lambda_E e^{\beta E} Z_{\mathrm{S}}^N\right\}\right] \\ \text{s.t.} \quad & \sum_{E\in\mathcal{E}_N} g_{N-1}(E-E_d)\lambda_E = p_d \qquad \forall d = 1,2,\ldots,D \\ & \lambda_E \geq 0 \qquad \forall E \in \mathcal{E}_N. \end{aligned} \tag{14}$$

To simplify the problem, we perform the change of variables

$$q_E = \lambda_E e^{\beta E} Z_{\mathrm{S}}^N. \tag{15}$$

We will also omit the term $k_{\mathrm{B}}T$ (being a positive constant) and drop the function log (given that log is a monotonically increasing function) obtaining

$$\min_q \quad \|q\|_\infty \tag{16}$$

$$\text{s.t.} \quad \sum_{E\in\mathcal{E}_N} \frac{g_{N-1}(E-E_d)}{Z_{\mathrm{S}}^N} e^{-\beta E} q_E = p_d \qquad \forall d = 1,2,\ldots,D \tag{17}$$

$$q_E \geq 0 \qquad \forall E \in \mathcal{E}_N, \tag{18}$$

where $\|\cdot\|_\infty$ is the infinity norm. Finally the minimization of the infinity norm can be linearized by introducing an auxiliary variable Q and additional constraints

$$\begin{aligned} &\min_{Q} Q \\ \text{s.t.} \quad \sum_{E\in\mathcal{E}_N} \frac{g_{N-1}(E-E_d)}{Z_{\mathrm{S}}^N} e^{-\beta E} q_E &= p_d && \forall d = 1,2,\ldots,D \\ q_E &\geq 0 && \forall E \in \mathcal{E}_N \\ q_E &\leq Q && \forall E \in \mathcal{E}_N \\ Q &\geq 0. \end{aligned} \tag{19}$$

As mentioned in the main text, linear optimization problems have been extensively studied and there exist numerical methods, such as the simplex algorithm, that allows one to solve this problem easily for arbitrary energy distributions. In the following section we will focus on the analytical solution for the qubit ($D = 2$) case, and then we will generalize these results to arbitrary dimensions.

Supplementary Note 2. Optimal solution for $D = 2$

When $D = 2$ the Hamiltonian of the system can be considered, without loss of generality, as $H_{\mathrm{S}} = E_0 |1\rangle\langle 1|$, where E_0 is the energy of the excited state. Thus, every block-diagonal state of S can written as $\rho = (1-p)|0\rangle\langle 0| + p|1\rangle\langle 1|$, with $p \in [0,1]$ the probability of being in the excited state.

The advantage of considering $D = 2$ is that there is a simple closed formula for the degeneracy of the N-partite energy levels in the set $\mathcal{E}_N = \{mE_0, \text{with } m = 0,1,\ldots,N\}$, that is given by

$$g_N(mE_0) = \binom{N}{m}, \quad m = 0,1,\ldots,N, \tag{20}$$

where $\binom{N}{m}$ is the binomial coefficient. Then, the constrains of Supplementary Equation (17) and Supplementary Equation (18) can easily be written as:

$$\sum_{m=0}^{N} \frac{1}{Z_{\mathrm{S}}^N} \binom{N}{m} e^{-\beta m E_0} q_m = \sum_{m=0}^{N} g_m q_m = 1, \tag{21}$$

$$\sum_{m=0}^{N} \frac{1}{Z_{\mathrm{S}}^N} \frac{m}{N} \binom{N}{m} e^{-\beta m E_0} q_m = \sum_{m=0}^{N} \frac{m}{N} g_m q_m = p, \tag{22}$$

where $g_m = \frac{1}{Z_{\mathrm{S}}^N}\binom{N}{m} e^{-\beta m E_0}$ and we have used the fact that $\binom{N-1}{m-1} = \frac{m}{N}\binom{N}{m}$.

The following Theorem characterizes the state $\rho_{\min}^{(N)}$ which minimizes the work of formation.

Theorem 1 (Exact solution, $D = 2$). Consider the optimization problem

$$\begin{aligned} &\min_{q\in\mathbb{R}^{N+1}} \quad \|q\|_\infty \\ \text{s.t.} \quad &\sum_{m=0}^{N} g_m q_m = 1 && (23) \\ &\sum_{m=0}^{N} m g_m q_m = Np && (24) \\ &q_m \geq 0, && (25) \end{aligned}$$

where $p \in [0,1]$, $N \in \mathbb{N}$ and g_m are non-negative real numbers. Then, there exists a unique solution and it is given by

$$q_m^* = \begin{cases} \gamma & \text{if } m \in U \\ 0 & \text{if } m \in L \\ s\gamma & \text{if } m \in (U\cup L)^c \end{cases} \tag{26}$$

where $\gamma \in \mathbb{R}_{>0}$, $s \in [0,1]$ and U, L are two different intervals of indexes such that $\#((U \cup L)^c) \leq 1$ (i.e. there is at most only one element not in either U or L). Moreover, if $(U \cup L)^c = \{m^*\}$ then $U = \{0, 1, \dots, m^* - 1\}$ and $L = \{m^* + 1, m^* + 2, \dots, N\}$, or $U = \{m^* + 1, m^* + 2, \dots, N\}$ and $L = \{0, 1, \dots, m^* - 1\}$; and γ and s are given by

$$\gamma = \frac{1}{s g_{m^*} + \sum_{m \in U} g_m} = \frac{m^* - Np}{\sum_{m \in U}(m^* - m) g_m} \quad , \quad s = \frac{1}{g_{m^*}} \frac{\sum_{m \in U}(Np - m) g_m}{m^* - Np}. \tag{27}$$

On the other hand, if $(U \cup L)^c = \emptyset$ then $s = 0$ and

$$\gamma = \frac{1}{\sum_{m \in U} g_m}. \tag{28}$$

Proof. Since $\|\cdot\|_\infty$ is a metric in $\mathbb{R}^{N+1}$ and the set of Supplementary Equation (23), Supplementary Equation (24) and Supplementary Equation (25) define a convex bounded set, there exists at least one solution to the problem. To see that the optimal solution is of the form of Supplementary Equation (26), we will show by contradiction that a smaller minimum cannot exist. First notice that the defining property of Supplementary Equation (26) states that q^*_m is either 0 or achieves its maximum (infinity norm) at all points except for at most a single one. Let's then assume that there is a different optimal solution $q^\dagger$ with smaller infinity norm such that it has at least a second non-zero component smaller than $\|q^\dagger\|_\infty$, i.e. there are indexes $a, b \in \{0, 1, \dots, N\}$ such that $0 < q^\dagger_a < \|q^\dagger\|_\infty$ and $0 < q^\dagger_b < \|q^\dagger\|_\infty$. Now we can define the sets $U^\dagger = \{m : q^\dagger_m = \|q^\dagger\|_\infty\}$ and $L^\dagger = \{m : q^\dagger_m = 0\}$. Since $q^\dagger$ satisfy Supplementary Equation (23) and Supplementary Equation (24):

$$\begin{aligned}
\sum_{m=0}^{N} g_m q^\dagger_m &= \|q^\dagger\|_\infty \sum_{m \in U^\dagger} g_m + \sum_{m \in (U^\dagger \cup L^\dagger)^c} g_m q^\dagger_m \\
&= (\|q^\dagger\|_\infty - \epsilon) \sum_{m \in U^\dagger} g_m + \sum_{m \in (U^\dagger \cup L^\dagger)^c} g_m q^\dagger_m + \epsilon \sum_{m \in U^\dagger} g_m \\
&= 1,
\end{aligned} \tag{29}$$

$$\begin{aligned}
\sum_{m=0}^{N} m g_m q^\dagger_m &= \|q^\dagger\|_\infty \sum_{m \in U^\dagger} m g_m + \sum_{m \in (U^\dagger \cup L^\dagger)^c} m g_m q^\dagger_m \\
&= (\|q^\dagger\|_\infty - \epsilon) \sum_{m \in U^\dagger} m g_m + \sum_{m \in (U^\dagger \cup L^\dagger)^c} m g_m q^\dagger_m + \epsilon \sum_{m \in U^\dagger} m g_m \\
&= Np,
\end{aligned} \tag{30}$$

where $0 < \epsilon < \|q^\dagger\|_\infty$. Let's see that in such a case there is another $q^{\dagger\dagger}$ with $\|q^{\dagger\dagger}\|_\infty < \|q^\dagger\|_\infty$, defined as

$$q^{\dagger\dagger}_m = \begin{cases} \|q^\dagger\|_\infty - \epsilon & \text{if } m \in U^\dagger \\ q^{\dagger\dagger}_a & \text{if } m = a \\ q^{\dagger\dagger}_b & \text{if } m = b \\ 0 & \text{if } m \notin U^\dagger \cup \{a, b\} \end{cases} \tag{31}$$

where $q^{\dagger\dagger}_a$, $q^{\dagger\dagger}_b$ and ϵ are constants to be determined satisfying the constrains. Notice that if $q^{\dagger\dagger}_a, q^{\dagger\dagger}_b < \|q^\dagger\|_\infty - \epsilon$, then $q^{\dagger\dagger}$ is a better minimum than $q^\dagger$. Additionally, $q^{\dagger\dagger}$ will satisfy the constrains of Supplementary Equation (23) and Supplementary Equation (24) if there are constants $q^{\dagger\dagger}_a, q^{\dagger\dagger}_b, \epsilon > 0$ such that

$$\begin{bmatrix} g_a & g_b \\ a g_a & b g_b \end{bmatrix} \begin{bmatrix} q^{\dagger\dagger}_a \\ q^{\dagger\dagger}_b \end{bmatrix} = \begin{bmatrix} g_a q^\dagger_a + g_b q^\dagger_b + \epsilon \sum_{m \in U^\dagger} g_m \\ a g_a q^\dagger_a + b g_b q^\dagger_b + \epsilon \sum_{m \in U^\dagger} m g_m \end{bmatrix}. \tag{32}$$

Since $g_a, g_b \neq 0$ and $a \neq b$, the first matrix is invertible and then

$$\begin{aligned}
\begin{bmatrix} q^{\dagger\dagger}_a \\ q^{\dagger\dagger}_b \end{bmatrix} &= \begin{bmatrix} q^\dagger_a \\ q^\dagger_b \end{bmatrix} + \epsilon \frac{(g_a g_b)^{-1}}{b - a} \begin{bmatrix} b g_b \sum_{m \in U^\dagger} - g_b \sum_{m \in U^\dagger} m g_m \\ g_a \sum_{m \in U^\dagger} m g_m - a g_a \sum_{m \in U^\dagger} g_m \end{bmatrix} \\
&\doteq \begin{bmatrix} q^\dagger_a \\ q^\dagger_b \end{bmatrix} + \epsilon \begin{bmatrix} s_a \\ s_b \end{bmatrix}.
\end{aligned} \tag{33}$$

Thus, since $0 < q_i^\dagger < \|q^\dagger\|_\infty$ $(i = a, b)$ and s_a, s_b do not depend on $q^{\dagger\dagger}$, there exists $\epsilon > 0$ with $0 < q_i^{\dagger\dagger} < \|q^\dagger\|_\infty - \epsilon$ $(i = a, b)$. As a consequence, $q^{\dagger\dagger}$ verifies Supplementary Equation (23), Supplementary Equation (24), Supplementary Equation (25) and $\|q^{\dagger\dagger}\|_\infty < \|q^\dagger\|_\infty$. Therefore, $q^\dagger$ cannot be the solution of the optimization problem, and then the optimal solution is the one given in Supplementary Equation (26).

On the other hand, the expressions of γ y s in Supplementary Equation (27) follow from the fact that

$$\begin{bmatrix} \sum_{m\in U} g_m & g_{m^*} \\ \sum_{m\in U} m g_m & m^* g_{m^*} \end{bmatrix} \begin{bmatrix} \gamma \\ s\gamma \end{bmatrix} = \begin{bmatrix} 1 \\ Np \end{bmatrix}, \tag{34}$$

where $(U \cup L)^c = \{m^*\}$. Now, for a fixed m^* let us demonstrate that either $U = \{0, 1, \ldots, m^* - 1\}$ and $L = \{m^*+1, m^*+2, \ldots, N\}$, or $U = \{m^*+1, m^*+2, \ldots, N\}$ and $L = \{0, 1, \ldots, m^*-1\}$. From Supplementary Equation (34) we have that

$$\gamma = \frac{m^* - Np}{\sum_{m\in U}(m^* - m) g_m}, \tag{35}$$

and for a fixed m^* and s, it is then clear that the way to minimize γ is by choosing U as a set of consecutive indexes as big as possible. Also, with U fixed the best strategy is to choose m^* as a consecutive index in order to ensure that γ will be positive and as small as possible.

It is worth noting that the cases $s = 0$ and $s = 1$ are equivalent with a proper redefinition of U. Indeed, whenever $s = 1$, we can define $U' = U \cup \{m^*\}$ and then $(U' \cup L)^c = \emptyset$, which by definition means that $s' = 0$. Conversely, if $s = 0$ (and therefore by definition $(U \cup L)^c = \emptyset$), we can take any element m^* in U (in this case U can never be empty, otherwise $q = 0$) and define $U' = U \setminus \{m^*\}$ which means that now $(U' \cup L)^c = \{m^*\}$ and $s' = 1$. For this reason, in the remainder of this text we will either use $s = 1$ or $s = 0$ (each with their own proper definition of U) for the same state depending on the convenience for the calculation at hand.

Corollary 1 (Theorem 1 in the main text). Given an integer N and a state ρ which satisfies $[\rho, H_{\mathrm{S}}] = 0$, there exists a subset of energies $\mathcal{E}_N^\rho \subseteq \mathcal{E}_N$, a constant $s \in (0, 1]$, and at most a single energy $\varepsilon \in \mathcal{E}_N$ such that the state $\rho_{\min}^{(N)}$ is defined by the distribution:

$$\lambda_E = \begin{cases} \frac{e^{-\beta E}}{\gamma} & \text{if } E \in \mathcal{E}_N^\rho \\ s\,\frac{e^{-\beta\varepsilon}}{\gamma} & \text{if } E = \varepsilon \\ 0 & \text{otherwise} \end{cases} \tag{36}$$

with γ a normalization constant. The work of formation and the extractable work of $\rho_{\min}^{(N)}$ are given by

$$\mathcal{W}_{\mathrm{form}}(\rho, N) = k_{\mathrm{B}} T \log\left[\frac{Z_{\mathrm{S}}^N}{\gamma}\right], \tag{37}$$

$$\mathcal{W}_{\mathrm{ext}}(\rho, N) = k_{\mathrm{B}} T \log\left[\frac{Z_{\mathrm{S}}^N}{Z}\right], \tag{38}$$

where Z is the partition function of a system in a thermal state at temperature T with spectrum given by the set $\mathcal{E}_N^\rho \cup \{\varepsilon\}$, and $\gamma = Z - (1 - s)\, g_N(\varepsilon) e^{-\beta\varepsilon}$.

Proof. It follows from Theorem 1 taking $q_E = \lambda_E e^{\beta E} Z_{\mathrm{S}}^N$, defining $\mathcal{E}_N^\rho = \{mE_0 : \text{with } m \in U\}$ and $\varepsilon = m^* E_0$ with m^* the only index in $(U \cup L)^c$ (if $(U \cap L)^c = \emptyset$, we redefine U as mentioned after the proof of Theorem 1 to have $(U \cup L)^c = \{m^*\}$ and $s = 1$). The c-work of formation is then given by

$$\begin{aligned} \mathcal{W}_{\mathrm{form}}(\rho, N) &= k_{\mathrm{B}} T \log \gamma^{-1} \\ &= -k_{\mathrm{B}} T \log\left[\sum_U g_m + s g_{m^*}\right] \\ &= -k_{\mathrm{B}} T \log\left[\frac{1}{Z_{\mathrm{S}}^N}\left(\sum_{U\cup\{m^*\}} \binom{N}{m} e^{-\beta m E_0} - (1-s) g_N(\varepsilon) e^{-\beta\varepsilon}\right)\right] \\ &= k_{\mathrm{B}} T \log\left[\frac{Z_{\mathrm{S}}^N}{\gamma}\right] \end{aligned} \tag{39}$$

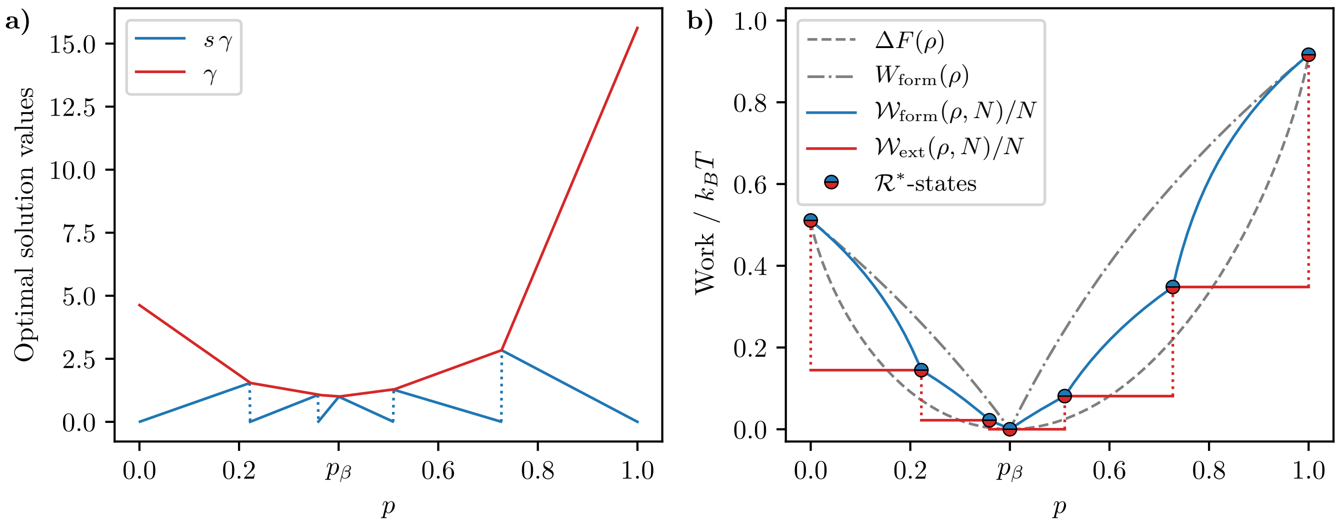

Supplementary Figure 1. **Optimal solution for the qubit case and $N = 3$ copies**. Panel (a) shows the optimal values of γ (red curve) and $s\gamma$ (blue curve) as a function of the local qubit excited state probability, p. The dotted lines indicate discontinuities that appear in $s\gamma$. Panel (b) shows the work of formation of a single copy $W_{\text{form}}(\rho)$ (gray dash-dotted line), the c-work of formation per copy $\mathcal{W}_{\text{form}}(\rho, N)/N$ (blue line), the extractable work from the correlated system per copy $\mathcal{W}_{\text{ext}}(\rho, N)/N$ (red line) and the standard thermodynamic free energy of the local system ΔF (dahsed gray line). The blue and red circles further indicate the location of the reversible states.

where Z is the partition function of the system restricted to the set of energies $\mathcal{E}_N^{\rho} \cup \{\varepsilon\}$, that is,

$$Z = Z_{\text{S}}^N \sum_{m \in \mathcal{E}_N^{\rho} \cup \{\varepsilon\}} g_m = \sum_{m \in \mathcal{E}_N^{\rho} \cup \{\varepsilon\}} \binom{N}{m} e^{-\beta m E_0}. \tag{40}$$

Supplementary Figure 1-a) illustrates the analytical solution for $N = 3$. There we show the dependence of γ and $s\gamma$ (Supplementary Equation (27)) with the local state $\rho = (1-p)\,|0\rangle\langle 0| + p\,|1\rangle\langle 1|$. It can been seen that $\gamma(p)$ is a piecewise linear function and that the break points coincide abruptly changes of s from $s \approx 0$ to 1. A similar discontinuity in the derivative of γ appears in the derivative of the c-work of formation (Supplementary Figure 1-b)).

Supplementary Note 3.   Bound on the amount of total correlations

We have shown that correlated states minimize the c-work of formation. Thus, the creation of correlations can be used to reduce this energetic cost with respect to the uncorrelated case. The question we address here is whether there is a bound on the amount of total correlations that can be developed while reducing this work cost. It this easy to show that total correlations

$$\mathcal{I}(\rho^{(N)}) > N\beta\left(W_{\text{form}}(\rho) - \Delta F(\rho)\right) \tag{41}$$

are necessarily costly, since they cannot be used to reduced the work of formation. In fact, if a multipartite state $\rho^{(N)}$ is created with total correlations $\mathcal{I}(\rho^{(N)})/N > (W_{\text{form}}(\rho) - \Delta F(\rho))\beta$, then $\mathcal{I}(\rho^{(N)})\,/\beta + N\Delta F(\rho) > N\,W_{\text{form}}(\rho)$. Recalling that $\Delta F(\rho^{(N)}) = \mathcal{I}(\rho^{(N)})\,/\beta + N\Delta F(\rho)$ and $W_{\text{form}}(\rho^{(N)}) \geq \Delta F(\rho^{(N)})$ then $W_{\text{form}}(\rho^{(N)}) > N\,W_{\text{form}}(\rho)$.

Supplementary Note 4.   Reversibility and $\mathcal{R}^*$-states

As mentioned in the main text, in general the transformations in the resource theory framework are not reversible, in the sense that the work of formation of a state is in general strictly larger than the extractable work [1]. This means that in general we will not be able to recover all the work invested in the creation of a state from thermal equilibrium. However, there is a particular set of states where reversible interconversion can be achieved.

Definition 3 (Reversible states). A state σ is reversible if its work of formation and extractable work coincide; i.e.

$$W_{\rm ext}(\sigma) = W_{\rm form}(\sigma). \tag{42}$$

We define as $\mathcal{R}$ the set of all reversible states.

While generic states are irreversible, there is a particularly simple family of reversible states which are thermal on a reduced support of the system.

Proposition 2 (non-trivial reversibles states). Let S be a system with Hamiltonian $H_{\rm S}$ and energy spectrum $\mathcal{E}_{\rm S}$. Let $\tau_{\rm S}$ be the Gibbs state of the system, which is characterized by the eigenvalues

$$\tau_{\rm S}(E,g) = \frac{1}{Z_{\rm S}} e^{-\beta E} \quad \forall g \leq g_{\rm S}(E), \tag{43}$$

where $Z_{\rm S}$ is the partition function and $g_{\rm S}(\cdot)$ the degeneracy. Let ρ be a state of S with $[\rho, H_{\rm S}] = 0$ and eigenvalues given by

$$\lambda(E,g) = \frac{1}{Z} \tau_{\rm S}(E,g) \mathbb{1}_{\{E\in\mathcal{E}\}} \quad \forall g \leq g_{\rm S}(E), \tag{44}$$

where $\mathcal{E} \subseteq \mathcal{E}_{\rm S}$ is a subset of allowed energies, $\mathbb{1}$ is the indicator function and Z is a normalization constant. Then the family of Rényi divergences $\{D_\alpha(\rho\|\tau_{\rm S})\}_{\alpha\geq 0}$ is independent of α. In particular, the state ρ is reversible.

Proof. It follows directly from the definition of the Rényi divergences [2] and the form of ρ. Given $\alpha \geq 0$

$$\begin{aligned} D_\alpha(\rho\|\tau_{\rm S}) &= \frac{1}{\alpha-1} \log \sum_{E\in\mathcal{E}_{\rm S}} \sum_{g=1}^{g_{\rm S}(E)} \lambda(E,g)^\alpha \tau_{\rm S}(E,g)^{1-\alpha} \\ &= \frac{1}{\alpha-1} \log \sum_{E\in\mathcal{E}} g_{\rm S}(E) Z^{-\alpha} \tau_{\rm S}(E,g) \\ &= \frac{1}{\alpha-1} \log Z^{-\alpha} \sum_{E\in\mathcal{E}} g_{\rm S}(E) \tau_{\rm S}(E,g) \\ &= \frac{1}{\alpha-1} \log Z^{1-\alpha} \\ &= -\log Z. \end{aligned} \tag{45}$$

In particular,

$$D_0(\rho\|\tau_{\rm S}) = \inf_{\alpha>0} D_\alpha(\rho\|\tau_{\rm S}) = D_\infty(\rho\|\tau_{\rm S}) = \sup_{\alpha>0} D_\alpha(\rho\|\tau_{\rm S}) = -\log Z \tag{46}$$

and therefore $W_{\rm ext}(\rho) = W_{\rm form}(\rho)$.

A. $\mathcal{R}^*$-states

As we will show, there exists a set of N-partite reversible states that are indeed solution to the minimization problem. The associated reduced states $\rho = (1-p)\,|0\rangle\langle 0| + p\,|1\rangle\langle 1|$, parametrized by the value of p, are defined as $\mathcal{R}^*$-states and in Supplementary Figure 1-a) and Supplementary Figure 1-b) are associated to the breaking points in the c-work of formation.

Definition 4 ($\mathcal{R}^*$-states). Given a number of copies N and a reduced state $\rho(p^*) = (1-p^*)\,|0\rangle\langle 0| + p^*\,|1\rangle\langle 1|$, we say that $\rho(p^*)$ is an $\mathcal{R}^*$-state if $\mathcal{W}_{\rm form}(\rho(p^*), N) = \mathcal{W}_{\rm ext}(\rho(p^*), N)$. We call $\mathcal{R}^*(N)$ the set of all $\mathcal{R}^*$-states for a given N.

The set $\mathcal{R}^*(N)$ can be easily characterized by the condition $s = 1$ (see Corollary 1).

Proposition 3. Given a system of N qubits, the set of $\mathcal{R}^*$-states is determined by the $2N+1$ states of the form

$$\rho(p_k^*) = (1-p_k^*)\,|0\rangle\langle 0| + p_k^*\,|1\rangle\langle 1| \tag{47}$$

where

$$p_k^* = -\frac{1}{NE_0} \frac{\partial}{\partial\beta} \log Z_k = \frac{\langle E\rangle_k}{NE_0}. \tag{48}$$

If $p_k^* \leq p_\beta$, $Z_k \equiv \sum_{m=0}^{k} \binom{N}{m} e^{-\beta m E_0}$ for $k = 0,..,N$; while if $p_k^* > p_\beta$, $Z_k \equiv \sum_{m=0}^{k} \binom{N}{N-m} e^{-\beta(N-m)E_0}$ for $k = 0,..,N-1$. Here the Gibbs state is $\rho(p_\beta)$.

From Supplementary Equation (27) it follows that the values p_k^* for which $\rho(p_k^*)$ is an $\mathcal{R}^*$-state satisfies

$$p_k^* = \frac{1}{N}\frac{\sum_{U_k} m g_m}{\sum_{U_k} g_m} = -\frac{1}{NE_0}\frac{\partial}{\partial\beta}\log Z_k \tag{49}$$

with $U_k = \{k, k+1, \ldots, N\}$ or $U_k = \{0, 1, \ldots, k\}$, with $k = 0, 1, \ldots, N$, depending on whether $p_k^* \leq p_\beta$ or $p_k^* \geq p_\beta$. Notice that in both cases $k = N$ coincides with the Gibbs state. Therefore the c-work of formation for these states is just

$$\mathcal{W}_{\text{form}}(\rho(p_k^*), N) = -k_{\text{B}}T\log\left[\frac{Z_k}{Z_{\text{S}}^N}\right]. \tag{50}$$

From the family of $\mathcal{R}^*$-states it is possible to recover the optimal solution given by Theorem 1 for each $p \in [0,1]$. Let us consider two different $\mathcal{R}^*$-states characterized by consecutive values p_k^*, p_{k+1}^*:

$$p_k^* = \frac{1}{N}\frac{\sum_{m=k}^{N} m g_m}{\sum_{m=k}^{N} g_m} \quad , \quad p_{k+1}^* = \frac{1}{N}\frac{\sum_{m=k+1}^{N} m g_m}{\sum_{m=k+1}^{N} g_m}, \tag{51}$$

and using the same change of variables that was done in Supplementary Equation (15) we can define

$$q_n^{*,k} = \frac{1}{Z_k}\mathbb{1}_{\{k\leq n\}} \quad , \quad q_n^{*,k+1} = \frac{1}{Z_{k+1}}\mathbb{1}_{\{k+1\leq n\}}, \tag{52}$$

where $\mathbb{1}$ is the indicator function. Given $p \geq p_\beta$, let k be such that $p_k^* < p < p_{k+1}^*$ and $x \in (0,1)$ such that $p = xp_k^* + (1-x)p_{k+1}^*$. For this value of p the optimal solution is as follows:

$$\begin{aligned} q_n &= \frac{k - Np}{\sum_{m=k+1}^{N}(k-m)g_m}\mathbb{1}_{\{k+1\leq n\}} + \frac{1}{g_k}\frac{\sum_{m=k+1}^{N}(Np-m)g_m}{\sum_{k=m+1}^{N}(k-m)g_m}\mathbb{1}_{\{k=n\}} \\ &= x\frac{1}{\sum_{m=k}^{N} g_m}\mathbb{1}_{\{k\leq n\}} + (1-x)\frac{1}{\sum_{m=k+1}^{N} g_m}\mathbb{1}_{\{k+1\leq n\}} \\ &= xq_n^{*,k} + (1-x)q_n^{*,k+1}. \end{aligned} \tag{53}$$

Therefore, using the same convex combination that relates the value p with its two nearest $\mathcal{R}^*$-states, p_k^* and p_{k+1}^*, the optimal solution for p can be written as a convex combination of the optimal solutions of p_k^* and p_{k+1}^*.

B. Density

Another useful property that the $\mathcal{R}^*$-states satisfy is that they are dense in the space of states in the following sense. Let us consider the union of all the states $\mathcal{R}^*(N)$,

$$\mathcal{R}^* = \bigcup_{N\in\mathbb{N}} \mathcal{R}^*(N). \tag{54}$$

Then, given $\epsilon > 0$, for each ρ there exists $\rho_\epsilon \in \mathcal{R}^*$ such that $\|\rho - \rho_\epsilon\|_1 < \epsilon$. To prove this, we are going to show that the spacing between two consecutive values p_k^* and p_{k+1}^* for which the state $\rho = (1-p)|0\rangle\langle 0| + p|1\rangle\langle 1|$ is an $\mathcal{R}^*$-state goes to zero as $\mathcal{O}(1/N)$. Since the expression for p_k^* includes the factors g_m, for large enough N we can make use of asymptotic estimations for the binomial coefficients. From the following lemma we can conclude the main result of this section.

Lemma 1 ([3]). Let X a random variable with $X \sim Bi(n,p)$, where $n \in \mathbb{N}$ and $p \in [0,1]$. The probability that $X \leq m$ is given by

$$B_m(n,p) = \sum_{j=0}^{m}\binom{n}{m}p^j q^{n-j} \tag{55}$$

with $q = 1 - p$. Then, for each $0 \leq m \leq np - \phi(n)$ it holds that

$$B_m(n,p) \approx \frac{1}{1-r}\binom{n}{m} p^m q^{n-m} \tag{56}$$

where $r = qm/(p(n+1-m))$ and $\phi(n) = \mathcal{O}(\sqrt{n})$.

Proposition 4 (Density). Let N be the number of correlated copies of the system and $\rho = (1-p)\,|0\rangle\langle 0| + p\,|1\rangle\langle 1|$ the reduced state. Given two consecutive values p_k^* and p_{k+1}^* associated to two $\mathcal{R}^*$-states such that $|k - Np_\beta| \geq \phi(n)$, where $\phi(n) = \mathcal{O}(\sqrt{n})$, it follows

$$N(p_{k+1}^* - p_k^*) \xrightarrow{N\to\infty} 1. \tag{57}$$

Proof. From Lemma 1 it follows

$$\begin{aligned}
\sum_{m=k}^{N} g_m &= \sum_{m=k}^{N} \frac{1}{Z_{\mathrm{S}}^N}\binom{N}{m} e^{-\beta m E_0} \\
&= \sum_{m=k}^{N} \binom{N}{m}\left(\frac{e^{-\beta E_0}}{Z_{\mathrm{S}}}\right)^m \left(\frac{1}{Z_{\mathrm{S}}}\right)^{N-m} \\
&= \sum_{m=0}^{N-m_0} \binom{N}{m}\left(\frac{1}{Z_{\mathrm{S}}}\right)^m \left(\frac{e^{-\beta E_0}}{Z_{\mathrm{S}}}\right)^{N-m} \\
&\approx \frac{1}{1-r}\binom{N}{k}\left(\frac{e^{-\beta E_0}}{Z_{\mathrm{S}}}\right)^k \left(\frac{1}{Z_{\mathrm{S}}}\right)^{N-k} \\
&= \frac{1}{1-r} g_k,
\end{aligned} \tag{58}$$

where $r = e^{-\beta E_0}(n-k)/(k+1)$. Then,

$$1 + \frac{g_k}{\sum_{m=k+1}^N g_m} \approx e^{\beta E_0}\frac{k+1}{N-k}. \tag{59}$$

On the other hand, we for $p_k^* > p_\beta$ we can write:

$$p_k^* = \frac{1}{N}\frac{\sum_{m=k}^N m\binom{n}{m} e^{-\beta m E_0}}{\sum_{m=k}^N \binom{n}{m} e^{-\beta m E_0}} = -\frac{1}{E_0}\frac{\partial}{\partial\beta}\log\sum_{m=k}^N \binom{n}{m} e^{-\beta m E_0}. \tag{60}$$

Finally,

$$\begin{aligned}
N(p_{k+1}^* - p_k^*) &= \frac{1}{E_0}\frac{\partial}{\partial\beta}\log\left[\frac{\sum_{m=k}^N g_m}{\sum_{m=k+1}^N g_m}\right] \\
&= \frac{1}{E_0}\frac{\partial}{\partial\beta}\log\left[1 + \frac{g_k}{\sum_{m=k+1}^N g_m}\right] \\
&\approx \frac{1}{E_0}\frac{\partial}{\partial\beta}\left[\beta E_0 + \log\frac{k+1}{N-k}\right] = 1,
\end{aligned} \tag{61}$$

where we use that the approximation of Supplementary Equation (59) holds for the derivative. This follows from the fact that both terms in Supplementary Equation (59) are monotones in β and k.

C. Quasi-thermal states

In Fig. 2(b) of the main text we can observe that there is a set of states for which the c-work of formation of N copies coincides with the work of formation of a single copy. Since these states are concentrated around the Gibbs

state of the system, we will denote them as quasi-thermal states. For a fixed value of N, let $p_-^*, p_+^* \in [0,1]$ be the values associated to $\mathcal{R}^*$-states given by

$$p_-^* = \frac{1}{N}\frac{\sum_{m=0}^{N-1} m g_m}{\sum_{m=0}^{N-1} g_m} = \frac{p_\beta - e^{\beta N E_0}/Z_\mathrm{S}^N}{1 - e^{\beta N E_0}/Z_\mathrm{S}^N}, \tag{62}$$

$$p_+^* = \frac{1}{N}\frac{\sum_{m=1}^{N} m g_m}{\sum_{m=1}^{N} g_m} = \frac{p_\beta}{1-g_0}. \tag{63}$$

Notice that the length of the interval $[p_-^*, p_+^*]$ decreases exponentially with the number of copies N.

Proposition 5 (quasi-thermal states). For each local state $\rho = (1-p)\,|0\rangle\langle 0| + p\,|1\rangle\langle 1|$, with $p \in [p_-^*, p_+^*]$, it holds

$$\mathcal{W}_\mathrm{form}(\rho, N) = W_\mathrm{form}(\rho). \tag{64}$$

Proof. The work of formation of the state $\rho_+ = (1-p_+^*)\,|0\rangle\langle 0| + p_+^*\,|1\rangle\langle 1|$ is

$$W_\mathrm{form}(\rho_+) = k_\mathrm{B}T \log\left(\max\{p_+^* e^{\beta E_0} Z_\mathrm{S}, (1-p_+^*) Z_\mathrm{S}\}\right) = k_\mathrm{B}T\log\left(p_+^* e^{\beta E_0} Z_\mathrm{S}\right) \tag{65}$$

where we use that $p_\beta < p_+^*$. On the other hand, the c-work of formation of N copies of ρ_+ is

$$\begin{aligned}
\mathcal{W}_\mathrm{form}(\rho_+, N) &= k_\mathrm{B}T\log\left(\frac{1}{\sum_{m=1}^{N} g_m}\right) \\
&= k_\mathrm{B}T\log\left(\frac{1}{1-g_0}\right) \\
&= k_\mathrm{B}T\log\left(\frac{p_+^*}{p_\beta}\right) \\
&= k_\mathrm{B}T\log\left(p_+^*(1+e^{\beta E_0})\right) \\
&= k_\mathrm{B}T\log\left(p_+^* e^{\beta E_0} Z_\mathrm{S}\right),
\end{aligned} \tag{66}$$

which coincides with Supplementary Equation (65). Without the term $k_\mathrm{B}T\log$, the c-work of formation is linear between p_β and p_+^*. Therefore, in light of Supplementary Equation (53) the same result is true for each $p \in [p_\beta, p_+^*]$. In the same way we can prove the proposition for $p \in [p_-^*, p_\beta]$.

Supplementary Note 5. Thermodynamic limit

In this section we focus on recovering the thermodynamic limit from the states that minimize the c-work of formation. We will show that for any reduced state ρ, when the number of copies N goes to infinity, we recover the classical result for the work of formation $\mathcal{W}_\mathrm{form}(\rho, N)/N \approx \Delta F$, i.e. the work of formation coincides with the standard free energy difference. Supplementary Figure 2-a) shows the correlated work of formation per copy $\mathcal{W}_\mathrm{form}/N$ for different states ρ. On the other hand, in Supplementary Figure 2-b) we show the c-work of formation for different numbers of copies N of the system as a function of p. We can see how the c-work of formation per copy converges asymptotically to the standard free energy difference.

Let's see how to analytically prove these results in the thermodynamic limit. Since the $\mathcal{R}^*$-states provide a complete characterization of the c-work of formation for each state and in addition they are dense, it is enough to prove the limit just for the $\mathcal{R}^*$-states. Every $\mathcal{R}^*$-state is of the form $\rho^* = (1-p^*)\,|0\rangle\langle 0| + p^*\,|1\rangle\langle 1|$, with

$$p^* = \frac{1}{N}\frac{\sum_{m\in U} m g_m}{\sum_{m \in U} g_m}. \tag{67}$$

Since U is an interval of consecutive energies and the median energy of the N copies is Np^*, we necessarily have $\lceil Np^* \rceil \in U$ or $\lfloor Np^* \rfloor \in U$. For simplicity, let us just consider that $Np^* \in U$ (the other case can be treated

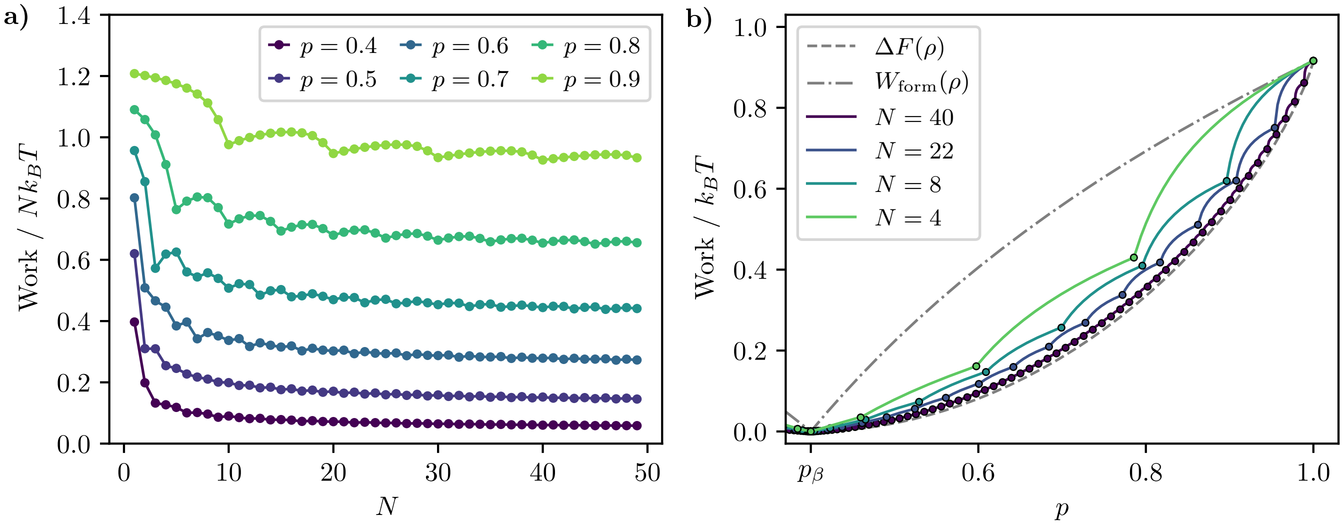

Supplementary Figure 2. **Convergence of the c-work of formation as the number of copies increases**. Panel (a) shows the c-work of formation $\mathcal{W}_{\rm form}(\rho, N)/N$ per copy for different local qubit states (parametrized by the excited state probability p) as a function of the number of copies N. We see how the work of formation converges towards the standard work of macroscopic thermodynamics $\Delta F(\rho)$ as N increases. Panel (b) shows the c-work of formation per copy $\mathcal{W}_{\rm form}(\rho, N)/N$ for different number of copies N as a function of the local qubit state (parametrized by the excited state probability p). We see how the correlated work of formation per copy converges to the standard free energy difference. The asymptotic value coincides with the standard work of macroscopic thermodynamics $\Delta F(\rho)$ and it is far from the work of formation of a single copy.

analogously). Then

$$\begin{aligned}
\mathcal{W}_{\rm form}(\rho^*, N) &= k_{\rm B}T \log\left(\frac{1}{\sum_{m\in U} g_m}\right) \\
&\leq k_{\rm B}T \log\left(\frac{1}{g_{Np^*}}\right) \\
&= k_{\rm B}T \log\left(\frac{Z_{\rm S}^N e^{\beta N p^* E_0}}{\binom{N}{Np^*}}\right) \\
&= Np^*E_0 + Nk_{\rm B}T\log Z_{\rm S} - k_{\rm B}T \log\binom{N}{Np^*}. \qquad (68)
\end{aligned}$$

Using Stirling's approximation for the binomial coefficients we have

$$\log\binom{N}{Np^*} = NS(p^*) + \mathcal{O}(\log N), \qquad (69)$$

where $S(\cdot)$ is the binary Shannon entropy. On one hand we have

$$\begin{aligned}
\mathcal{W}_{\rm form}(\rho^*, N) &\leq Np^*E_0 - k_{\rm B}TNS(p^*) + k_{\rm B}T\log Z_{\rm S} + \mathcal{O}(\log N) \\
&= N\left[F(\rho^*) - F(\tau)\right] + \mathcal{O}(\log N) \\
&= N\Delta F(\rho^*) + \mathcal{O}(\log N). \qquad (70)
\end{aligned}$$

On the other hand, it is easy to see that $N\Delta F(\rho^*) \leq \mathcal{W}_{\rm form}(\rho^*, N)$. Since $\rho_{\rm min}^{(N)}$ is reversible and due to the subadditivity of entropy we have

$$\begin{aligned}
\mathcal{W}_{\rm form}(\rho^*, N) &= \Delta F(\rho_{\rm min}^{(N)}) \\
&= {\rm tr}[H_{\rm S}^{\otimes N}\rho_{\rm min}^{(N)}] - k_{\rm B}TS(\rho_{\rm min}^{(N)}) + k_{\rm B}T\log Z_{\rm S}^N \\
&\geq N\,{\rm tr}[H_{\rm S}\rho^*] - Nk_{\rm B}TS(\rho^*) + Nk_{\rm B}T\log Z_{\rm S} \\
&= N\Delta F(\rho^*). \qquad (71)
\end{aligned}$$

Finally we have that the c-work of formation per copy converges to the standard free energy difference

$$\Delta F(\rho^*) \leq \frac{\mathcal{W}_{\text{form}}(\rho^*, N)}{N} \leq \Delta F(\rho^*) + \mathcal{O}\left(\frac{\log N}{N}\right). \tag{72}$$

To fully recover the expected thermodynamic limit would furthermore require that the correlations vanish as $N \to \infty$. We can compute the generalized mutual information between the correlated state $\rho_{\min}^{(N)}$ and the product state $\rho^{\otimes N}$,

$$\mathcal{I}\left(\rho_{\min}^{(N)}\right) = D_1\left(\rho_{\min}^{(N)} \| \rho^{*\otimes N}\right) = NS(\rho^*) - S(\rho_{\min}^{(N)}). \tag{73}$$

From the reversibility of the $\mathcal{R}^*$-states it is possible to rewrite the mutual information as a function of the c-work of formation $\mathcal{W}_{\text{form}}$ and the standard free energy difference ΔF,

$$\begin{aligned}
\mathcal{I}\left(\rho_{\min}^{(N)}\right) &= \beta[F(\rho^{(N)}) - NF(\rho^*)] \\
&= \beta[\Delta F(\rho^{(N)}) - N\Delta F(\rho^*)] \\
&= \beta[\mathcal{W}_{\text{form}}(\rho^*, N) - N\Delta F(\rho^*)] = \mathcal{O}(\log N).
\end{aligned} \tag{74}$$

We can then conclude that the amount of correlations scales as $\log N$ and therefore the correlations per copy vanish, thus the subsystems are weakly correlated in the macroscopic limit.

Supplementary Note 6. Generalization to systems of arbitrary dimension

In this section we will generalize the above results to systems of arbitrary finite dimension D. As we mentioned before, the analytical characterization of the minimization problem is quite difficult and requires the analytic expression of the degeneracy $g_N(E)$ for each energy E of the N-partite system, which cannot be known for arbitrary Hamiltonians. However, it can be proven that the optimal solution has the same form as the one given in Theorem 1. Furthermore, the existence of quasi-thermal states, their reversibility, density and the thermodynamic limit still hold for all D.

Let us consider the optimization problem presented in Supplementary Equation (16), Supplementary Equation (17) and Supplementary Equation (18) for systems of dimension D

$$\begin{aligned}
&\min_q \quad \|q\|_\infty \\
\text{s.t.} \quad &\sum_{E\in\mathcal{E}_N} \frac{g_{N-1}(E-E_d)}{Z_{\text{S}}^N} e^{-\beta E} q_E = p_d \qquad \forall d = 1, 2, \ldots, D \\
&q_E \geq 0 \qquad \forall E \in \mathcal{E}_N.
\end{aligned} \tag{75}$$

Notice that the form of the optimal solution introduced in Theorem 1 can be written as

$$q_i = \gamma \mathbb{1}_{\{i\in U\}} + s\gamma \mathbb{1}_{\{i=m^*\}}. \tag{76}$$

Let $A \in \mathbb{R}^{D\times M}$ (where $M = \#(\mathcal{E}_N)$ is the cardinality of the set of energies of the N-partite system) be the matrix where each row corresponds to an energy E_d of a single system and each column to an energy E of the N-partite system, and the matrix elements are equal to $g_{N-1}(E - E_d)e^{-\beta E}/Z_{\text{S}}^N$. The following theorem proves the natural generalization of Supplementary Equation (76), where now the optimal solution either realizes its infinity norm or it is equal to zero except in at most $D - 1$ points.

Theorem 2. Consider the optimization problem given by

$$\begin{aligned}
\min_{q\in\mathbb{R}^M} \quad &\|q\|_\infty \\
\text{s.t.} \quad &Aq = p \\
&q \geq 0
\end{aligned} \tag{77}$$

where $p \in \mathbb{R}^D_{\geq 0}$ and $A \in \mathbb{R}^{D\times M}_{\geq 0}$, such that there is at least one feasible solution to the constrains. Then, there exists a solution, not necessarily unique, of the form

$$q_i = \gamma \mathbb{1}_{\{i\in U\}} + \sum_{j=1}^{D-1} \gamma s_j \mathbb{1}_{\{i=n_j\}} \tag{78}$$

where $s_1, s_2, \ldots, s_{D-1} \in [0, 1]$; $\gamma \in \mathbb{R}_{>0}$; U is a set of indexes where q realizes its infinity norm; and $n_1, n_2, \ldots, n_{D-1} \in \{1, 2, 3, \ldots, M\}$.

Proof. Let $\mathcal{A}$ be the set of optimal solutions of Supplementary Equation (77). Since $\|\cdot\|_\infty$ is a distance and the constrains $Aq = p$ and $q \geq 0$ define a convex, bounded and closed set in $\mathbb{R}^M$, it follows that there is at least one solution and then $\mathcal{A}$ is non-empty. For each q we define

$$M(q) = \{i : q_i \neq 0 \wedge q_i \neq \|q\|_\infty\}\,, \tag{79}$$

and for each p let us consider the quantity

$$m(p) = \min\Big\{|M(q)| : q \text{ is a solution of Supplementary Equation (77)}\Big\}. \tag{80}$$

Then, the main statement of the Theorem is equivalent to proving that $m(p) \leq D-1$ for every p. Let $q^\dagger \in \mathcal{A}$ be such that $|M(q^\dagger)| = m(p)$ and assume that $m(p) \geq D$. Additionally, let $\{i_1, i_2, \ldots, i_D\} \subset M(q^\dagger)$ and consider the matrix

$$A_M = \left[\begin{array}{c|c|c|c} a_{i_1} & a_{i_2} & \cdots & a_{i_D} \end{array}\right], \tag{81}$$

where $a_{i_1}, \ldots, a_{i_D}$ are the columns $i_1, \cdots, i_D$ of the matrix A. We will now show that A_M cannot be invertible. If A_M were invertible, then it would be possible to explicitly construct a solution $q^{\dagger\dagger}$ with $\|q^{\dagger\dagger}\|_\infty < \|q^\dagger\|_\infty$ and given by

$$q^{\dagger\dagger}_m = \begin{cases} \|q^*\|_\infty - \epsilon & \text{if } q^\dagger_m = \|q^\dagger\|_\infty \\ x_1 & \text{if } m = i_1 \\ \vdots & \\ x_D & \text{if } m = i_D \\ q^\dagger_m & \text{otherwise,} \end{cases} \tag{82}$$

where $\epsilon > 0$ and $x_1, \ldots, x_D$ are chosen such that the constrains are satisfied and $x_1, \ldots, x_D < \|q^\dagger\|_\infty - \epsilon$. $q^{\dagger\dagger}$ is a feasible solution if

$$\begin{aligned} p = Aq^{\dagger\dagger} &= A_U \begin{bmatrix} \|q^*\|_\infty - \epsilon \\ \vdots \\ \|q^*\|_\infty - \epsilon \end{bmatrix} + A_M \begin{bmatrix} x_1 \\ \vdots \\ x_D \end{bmatrix} \\ &= Aq^\dagger + A_M \begin{bmatrix} x_1 - q^\dagger_{i_1} \\ \vdots \\ x_D - q^\dagger_{i_D} \end{bmatrix} - \epsilon \sum_{m \in U} a_m, \end{aligned} \tag{83}$$

where A_U is the restriction of A to the columns a_m for which $q^\dagger_m = \|q^\dagger\|_\infty$. Since $Aq^\dagger = p$ and assuming that A_M is invertible, then

$$\begin{bmatrix} x_1 - q^\dagger_{i_1} \\ \vdots \\ x_D - q^\dagger_{i_D} \end{bmatrix} = \epsilon A_M^{-1} \sum_{m \in U} a_m. \tag{84}$$

Since the right side of Supplementary Equation (84) is fixed and $0 < q^\dagger_{i_j} < \|q^\dagger\|_\infty$, it follows that there exists $\epsilon > 0$ small enough such that $0 < x_i < \|q^\dagger\|_\infty$. Then $\|q^{\dagger\dagger}\|_\infty = \|q^\dagger\|_\infty - \epsilon < \|q^\dagger\|_\infty$ and $q^\dagger$ cannot be an optimal solution, which contradicts our hypothesis. Therefore A_M is not invertible.

Since A_M is not invertible, there exists a non-trivial solution of the equation $A_M x = 0$. Then, it is possible to define a new solution to $Aq = p$ in the following way

$$q^{\dagger\dagger}_m = \begin{cases} \|q^*\|_\infty & \text{if } q^\dagger_m = \|q^\dagger\|_\infty \\ q^\dagger_m + \delta x_1 & \text{if } m = i_1 \\ \vdots & \\ q^\dagger_m + \delta x_D & \text{if } m = i_D \\ q^\dagger_m & \text{elsewhere.} \end{cases} \tag{85}$$

Then it is immediate that there exists $\delta > 0$ and an index i_k such that $q^{\dagger\dagger}_{i_k} = 0$ or $q^{\dagger\dagger}_{i_k} = \|q^\dagger\|_\infty$ and $0 < q^{\dagger\dagger}_m < \|q^\dagger\|_\infty$ for all $m \neq i_k$. Thus, $M(q^{\dagger\dagger}) = m(p) - 1$, which contradicts the definition of $m(p)$. Finally we conclude that $m(p) \leq D-1$ for all p.

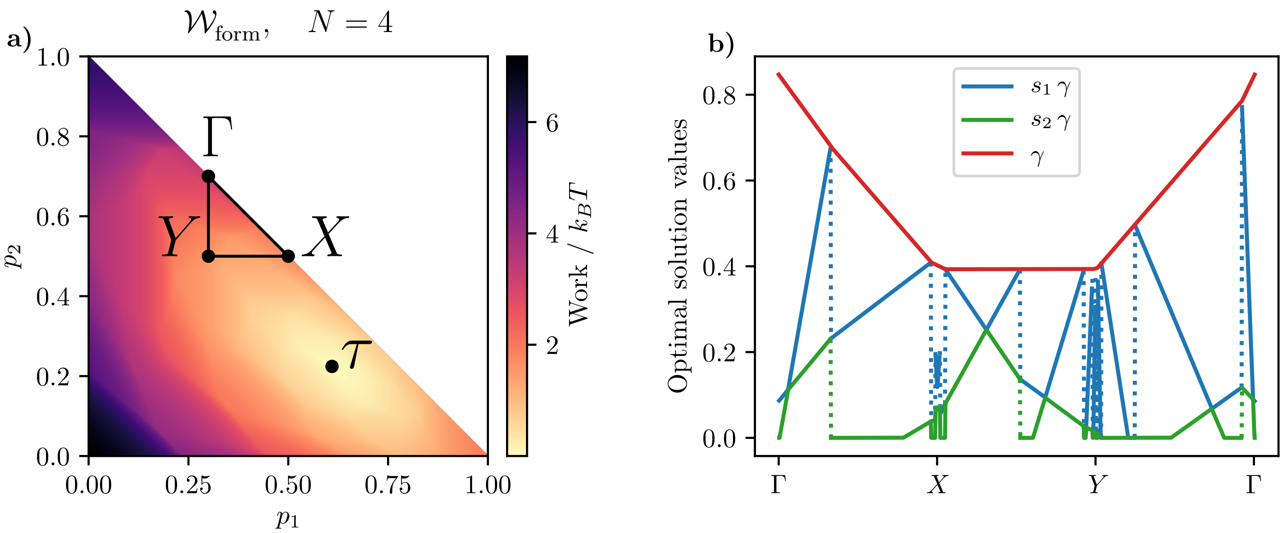

Supplementary Figure 3. **Optimal solution for the $D = 3$ case**. In panel (a) we show the correlated work of formation $\mathcal{W}_{\text{form}}$ for $N = 4$ copies of a system of dimension $D = 3$ for every reduced local state of the system. p_1, p_2 and p_3 correspond to the occupation of the energy levels E_1, E_2 and E_3, respectively. Panel (b) shows the values of γ, $s_1\gamma$ and $s_2\gamma$ of the optimal solution given by Theorem 2 for a particular path between states $\Gamma \to X \to Y \to \Gamma$.

As in the case of $D = 2$, Theorem 2 claims that the state that minimizes the work of formation is a renormalization of the Gibbs state over a restricted subset energies, except for at most $D - 1$ energy levels that escape the rule. Supplementary Figure 3 shows the value of the c-work of formation for $D = 3$ obtained numerically for each $p = (p_1, p_2, p_3)$, and the values of γ, $s_1\gamma$ and $s_2\gamma$ for a particular path of states.

A. $\mathcal{R}^*$-states

Here we will show the generalization of the $\mathcal{R}^*$-states to arbitrary dimension D. From now on, for simplicity, we will index the coordinates of q and the columns of A using the associated energy E of the N-partite system. We define the set of $\mathcal{R}^*$-states as the solutions of the optimization problem of Supplementary Equation (77) which are characterized by a subset of energies $\mathcal{E} \subset \mathcal{E}_N$ and which take the form

$$(q^*_{\mathcal{E}})_E = \gamma_{\mathcal{E}} \mathbb{1}_{\{E \in \mathcal{E}\}}, \tag{86}$$

where

$$\gamma_{\mathcal{E}} = \frac{1}{\left\| \sum_{E \in \mathcal{E}} a_E \right\|_1}. \tag{87}$$

An equivalent definition of the $\mathcal{R}^*$-states is given by the solutions that appear in Theorem 2 which also satisfy $s_1 = \ldots = s_{D-1} = 0$. Each $\mathcal{R}^*$-state of the form given by Supplementary Equation (86) has an associated vector $p^*_{\mathcal{E}}$ for which $q^*_{\mathcal{E}}$ is solution of $Aq^*_{\mathcal{E}} = p^*_{\mathcal{E}}$ and q^* is optimum, given by

$$p^*_{\mathcal{E}} = \frac{1}{\left\| \sum_{E \in \mathcal{E}} a_E \right\|_1} \sum_{E \in \mathcal{E}} a_E. \tag{88}$$

Notice that Proposition 2 is valid even for systems of arbitrary dimension D. Then it follows that the states defined in Supplementary Equation (86) are also reversible. On the other hand, for $D = 2$, the unique subset of energies $\mathcal{E}$ for which we find $\mathcal{R}^*$-states, are $\{E : E \geq kE_0, k = 0, 1, \ldots, N\}$ and $\{E : E \leq kE_0, k = 0, 1, \ldots, N\}$. However, for $D > 2$ and arbitrary energies $E_1, E_2, \ldots, E_D$ it is not possible to characterize the sets $\mathcal{E}$ and therefore we cannot find an analytical expression for the $\mathcal{R}^*$-states. However, we can give an implicit construction of them.

Proposition 6. Consider N copies of a system of dimension D and let $p \in \mathbb{R}^D$ be the occupation of the reduced state ρ of the system. Then, there exists a state of the N copies characterized by a vector $q(p)$ that minimizes the work of

formation such that there are $p_1^*, p_2^*, \dots, p_D^* \in \mathbb{R}^D$ probability vectors associated with $\mathcal{R}^*$-states $q(p_1^*), q(p_2^*), \dots, q(p_D^*)$ and

$$p = \lambda_1 p_1^* + \lambda_2 p_2^* + \ldots + \lambda_D p_D^*, \tag{89}$$

$$q(p) = \lambda_1 q(p_1^*) + \lambda_2 q(p_2^*) + \ldots + \lambda_D q(p_D^*), \tag{90}$$

where $\lambda_i \in \mathbb{R}_{\geq 0}$ with $\lambda_1 + \lambda_2 + \ldots + \lambda_D = 1$.

Proof. Let $s_1, s_2, \dots, s_{D-1}$ be ordered from highest to lowest and with associated energies $\varepsilon_1, \varepsilon_2, \dots, \varepsilon_{D-1}$, respectively. Now we denote by $\mathcal{E}_j$ the set of the first j energies $\varepsilon_1, \varepsilon_2, \dots, \varepsilon_j$. Then:

$$\begin{aligned} q_E &= \gamma_{\mathcal{E}} \mathbb{1}_{\{E\in\mathcal{E}\}} + \sum_{j=1}^{D-1} \gamma s_j \mathbb{1}_{\{E=\varepsilon_j\}} \\ &= \gamma_{\mathcal{E}}(1-s_1)\mathbb{1}_{\{E\in\mathcal{E}\}} \\ &+ \gamma_{\mathcal{E}}(s_1-s_2)\mathbb{1}_{\{E\in\mathcal{E}\}\cup\{E\in\mathcal{E}_1\}} \\ &\quad \vdots \\ &+ \gamma_{\mathcal{E}}(s_{D-2}-s_{D-1})\mathbb{1}_{\{E\in\mathcal{E}\}\cup\{E\in\mathcal{E}_{D-2}\}} \\ &+ \gamma_{\mathcal{E}} s_{D-1}\mathbb{1}_{\{E\in\mathcal{E}\}\cup\{E\in\mathcal{E}_{D-1}\}} \\ &= \lambda_1 (q_{\mathcal{E}}^*)_E + \lambda_2 (q_{\mathcal{E}\cup\mathcal{E}_1}^*)_E + \ldots + \lambda_D (q_{\mathcal{E}\cup\mathcal{E}_{D-1}}^*)_E, \end{aligned} \tag{91}$$

where $\lambda_1, \lambda_2, \dots, \lambda_D$ are all positive values that satisfy $\lambda_1 + \lambda_2 + \ldots + \lambda_D = 1$ and are given by

$$\lambda_1 = \gamma_{\mathcal{E}}(1-s_1) \left\| \sum_{E\in\mathcal{E}} a_E \right\|_1 \quad , \quad \lambda_D = \gamma_{\mathcal{E}} s_{D-1} \left\| \sum_{E\in\mathcal{E}\cup\mathcal{E}_{D-1}} a_E \right\|_1 \quad \text{and} \tag{92}$$

$$\lambda_j = \gamma_{\mathcal{E}} \left(s_{j-1} - s_j\right) \left\| \sum_{E\in\mathcal{E}\cup\mathcal{E}_{j-1}} a_E \right\|_1 \quad \text{for } j = 2, 3, \dots, D-1. \tag{93}$$

Then, each solution in Theorem 2 can be written as a linear convex sum of states of the form Supplementary Equation (86)

$$q = \lambda_1 q_{\mathcal{E}}^* + \lambda_2 q_{\mathcal{E}\cup\mathcal{E}_1}^* + \ldots + \lambda_D q_{\mathcal{E}\cup\mathcal{E}_{D-1}}^*. \tag{94}$$

What remains is to prove that each term of the convex sum in Supplementary Equation (94) is associated with an $\mathcal{R}^*$-state. Let's assume that $q_{\mathcal{E}}^*$ is not an $\mathcal{R}^*$-state. Then there exists $\tilde{q}$ with $\|\tilde{q}\|_\infty < \|q_{\mathcal{E}}^*\|_\infty$ and $A\tilde{q} = Aq_{\mathcal{E}}^*$. It is then clear that

$$q^\dagger = \lambda_1 \tilde{q} + \lambda_2 q_{\mathcal{E}\cup\mathcal{E}_1}^* + \ldots + \lambda_D q_{\mathcal{E}\cup\mathcal{E}_{D-1}}^* \tag{95}$$

satisfies $Aq^\dagger = Aq$. On the other hand,

$$\begin{aligned} \|q^\dagger\|_\infty &\leq \lambda_1 \|\tilde{q}\|_\infty + \ldots + \lambda_D \|q_{\mathcal{E}\cup\mathcal{E}_{D-1}}^*\|_\infty \\ &< \lambda_1 \|q_{\mathcal{E}}^*\|_\infty + \ldots + \lambda_D \|q_{\mathcal{E}\cup\mathcal{E}_{D-1}}^*\|_\infty \\ &= \lambda_1 \frac{1}{\left\|\sum_{E\in\mathcal{E}} a_E\right\|_1} + \ldots + \lambda_D \frac{1}{\left\|\sum_{E\in\mathcal{E}\cup\mathcal{E}_{D-1}} a_E\right\|_1} \\ &= \gamma_{\mathcal{E}} = \|q\|_\infty, \end{aligned} \tag{96}$$

that is $\|q^\dagger\| < \|q\|_\infty$, which contradicts the definition of q. In the same way we deduce that all $q_{\mathcal{E}\cup\mathcal{E}_1}^*, \dots, q_{\mathcal{E}\cup\mathcal{E}_{D-1}}^*$ are $\mathcal{R}^*$-states.

The existence of quasi-thermal states can be obtained numerically. Just like in the $D = 2$ case, there is a region surrounding the Gibbs state of the system for which the c-work of formation of N copies coincides with the work of formation of a single copy (see for example Supplementary Figure 3).

B. Density of reversible states

Without an analytic expression for the $\mathcal{R}^*$-states and their respective occupation levels p^*, we cannot prove rigorously that the spacing between the vales of p^* goes to zero when $N \to \infty$. Notice that even for the simplest case $D = 2$ it was necessary to use an asymptotic approximation of the binomial coefficients. However, we now present an argument for assuming that the distance between the terms that appear in Supplementary Equation (90) (which generalize p_k^* and p_{k+1}^* of Proposition 4) should converge to zero when N goes to infinity. In the worst case scenario, the distance in $\|\cdot\|_1$ between close values of p^* is

$$\begin{aligned}\left\|p^*_{\mathcal{E}\cup\mathcal{E}_{D-1}} - p^*_{\mathcal{E}}\right\|_1 &= \left\| \frac{1}{\left\|\sum_{E\in\mathcal{E}\cup\mathcal{E}_{D-1}} a_E\right\|_1} \sum_{E\in\mathcal{E}\cup\mathcal{E}_{D-1}} a_E - \frac{1}{\left\|\sum_{E\in\mathcal{E}} a_E\right\|_1} \sum_{E\in\mathcal{E}} a_E \right\|_1 \\ &= \left\| \frac{\sum_{E\in\mathcal{E}_{D-1}} a_E}{\left\|\sum_{E\in\mathcal{E}\cup\mathcal{E}_{D-1}} a_E\right\|_1} - \frac{\left\|\sum_{E\in\mathcal{E}_{D-1}} a_E\right\|_1 \sum_{E\in\mathcal{E}} a_E}{\left\|\sum_{E\in\mathcal{E}\cup\mathcal{E}_{D-1}} a_E\right\|_1 \left\|\sum_{E\in\mathcal{E}} a_E\right\|_1} \right\|_1 \\ &\leq 2\frac{\left\|\sum_{E\in\mathcal{E}_{D-1}} a_E\right\|_1}{\left\|\sum_{E\in\mathcal{E}\cup\mathcal{E}_{D-1}} a_E\right\|_1}, \end{aligned} \tag{97}$$

where we use that all a_E have non-negative entries. Notice that the amount of terms in the numerator is at most $D-1$, while the number of terms in the denominator is as large as the size of the set $\mathcal{E}$ of energies that defines the $\mathcal{R}^*$-state. It is reasonable to assume that the size of $\mathcal{E}$ increases with the number of copies and then

$$\lim_{N\to\infty} \left\|p^*_{\mathcal{E}\cup\mathcal{E}_{D-1}} - p^*_{\mathcal{E}}\right\|_1 = 0. \tag{98}$$

Instead of proving the above statement, that would be the direct analog of the proof provided for the 2-dimensional case for arbitrary dimension, we will instead prove that general reversible states are dense, in the sense that given a probability vector $p \in \mathbb{R}^D$, then there is a sequence of reversible states $\{\rho^{(N)}_{\text{rev}}\}_N$ such that

$$\lim_{N\to\infty} \max_{j=1,2,\dots,N} \left\| \text{tr}_{-j}\left[\rho^{(N)}_{\text{rev}}\right] - \rho(p)\right\|_1 = 0, \tag{99}$$

with $\rho(p) = \sum_{d=1}^D p_d \left|E_d\middle\rangle\middle\langle E_d\right|$. Unlike before, we will not require that these states also be a solution to the work of formation optimization problem. Nonetheless, this weaker result will still be enough to prove the thermodynamic limit.

Given the probability vector $p \in \mathbb{R}^D$, for each $d = 1, 2, \dots, D$ we define

$$n_i^d = \lfloor p_i N \rfloor, \qquad \text{for } i \in \{1, \dots, D\} \setminus \{d\}, \tag{100}$$

$$n_d^d = N - \sum_{i\neq d} n_i^d = N - \sum_{i\neq d} \lfloor p_i N \rfloor, \tag{101}$$

and the following states σ_d

$$\sigma_d = \frac{1}{C_d} \sum_{\text{permutations}} \underbrace{|E_1\rangle\langle E_1| \otimes \dots \otimes |E_1\rangle\langle E_1|}_{n_1^d \text{ times}} \otimes \dots \otimes \underbrace{|E_D\rangle\langle E_D| \otimes \dots \otimes |E_D\rangle\langle E_D|}_{n_D^d \text{ times}}, \tag{102}$$

where the sum runs over all the possible permutations of the N copies, and C_d is a normalization constant given by

$$C_d = \frac{N!}{(n_1^d)!(n_2^d)!\dots(n_D^d)!}. \tag{103}$$

Notice that the states σ_d are eigenstates of the total Hamiltonian with energy $E^{(d)} = n_1^d E_1 + \dots + n_D^d E_D$, and their reduced state is

$$\text{tr}_{-j}(\sigma_d) = \sum_{i=1}^D \frac{n_i^d}{N} |E_i\rangle\langle E_i| \quad \forall j = 1, 2, \dots, N. \tag{104}$$

which is clear that converges to $\rho(p)$ as $N \to \infty$. Additionally, it is easy to see that all σ_d are reversible, since only a single energy level is equally populated for each d. Thus, we have defined a family of reversible states whose reduced state is equal to $\rho(p)$ (in the case where $Np_i \in \mathbb{N}$, otherwise it converges to $\rho(p)$). In the next section we will show that while for finite N these reversible states are not optimal (in the sense that they do not minimize the c-work of formation for finite N) their work of formation per copy converges to the standard free energy difference in the thermodynamic limit.

Before showing how one can recover standard results in the thermodynamic limit, we will first make a connection between these states and the optimal ones through the following lemma.

Lemma 2. There is at least one of the energies $E^{(d)}$ that belongs to the support $\mathcal{E}_N$ of energies of the $\mathcal{R}^*$-state $\rho_{\min}^{(N)}$.

Proof. Let's assume that $E^{(d)} \notin \mathcal{E}_N$ for all $d = 1, 2, \ldots, D$. Consider the state given by

$$\sigma = \sum_{d=1}^{D} \lambda_d \, \sigma_d \quad \text{with} \quad \lambda_d = \frac{Np_d - \lfloor Np_d \rfloor}{\sum_{i=1,\ldots,D} Np_i - \lfloor Np_i \rfloor}. \tag{105}$$

In the case where $Np_d \in \mathbb{N}$ for all $d = 1, 2, \ldots, D$, we define $\sigma = \sigma_1$. Since $\mathrm{tr}_{-j}(\sigma) = \rho(p)$, given $\epsilon > 0$ we can define a new state $\rho^{(N)}$ locally equivalent to $\rho(p)$:

$$\rho^{(N)} = (1-\epsilon)\rho_{\min}^{(N)} + \epsilon\sigma. \tag{106}$$

Let's see now that the work of formation of $\rho^{(N)}$ is lower than the work of formation of $\rho_{\min}^{(N)}$. Since by hypothesis σ and $\rho_{\min}^{(N)}$ have disjoint support, the work of formation of $\rho^{(N)}$ is

$$W_{\text{form}}(\rho^{(N)}) = k_{\text{B}} T \log \max \left\{ (1-\epsilon) \frac{\lambda_E Z_{\text{S}}^N}{e^{-\beta E}}, \frac{\epsilon \lambda_1 Z_{\text{S}}^N}{C_1 e^{-\beta E^{(1)}}}, \ldots, \frac{\epsilon \lambda_D Z_{\text{S}}^N}{C_D e^{-\beta E^{(D)}}} \right\}, \tag{107}$$

where λ_E are the eigenvalues of $\rho_{\min}^{(N)}$. Then, there exists $\epsilon > 0$ small enough such that the maximum is realized in the first element and then

$$\begin{aligned} W_{\text{form}}(\rho^{(N)}) &= k_{\text{B}} T \log \left[(1-\epsilon) \frac{\lambda_E Z_{\text{S}}^N}{e^{-\beta E}} \right] \\ &= W_{\text{form}}(\rho_{\min}^{(N)}) + k_{\text{B}} T \log(1-\epsilon) < W_{\text{form}}(\rho_{\min}^{(N)}), \end{aligned} \tag{108}$$

which is a contradiction that comes from assuming that $E^{(d)} \notin \mathcal{E}_N$ for all $d = 1, 2, \ldots, D$.

In the case where $Np_i \in \mathbb{N}$, this lemma states that the mean energy $Np_1 E_1 + \ldots + Np_D E_D$ is part of the support of energies of the optimal state $\rho_{\min}^{(N)}$. In the other cases, we can find an energy as close as we want to the mean energy as $N \to \infty$.

C. Thermodynamic limit

We will first show that as N increases the c-work of formation per copy $\mathcal{W}_{\text{form}}(\rho, N)/N$ converges to the standard free energy difference for systems of arbitrary dimension D, that is,

$$\frac{\mathcal{W}_{\text{form}}(\rho, N)}{N} \xrightarrow{N \to \infty} \Delta F(\rho). \tag{109}$$

Moreover, the convergence rate is $\mathcal{O}(\log N/N)$. Just like as in the case $D = 2$, let us consider the $\mathcal{R}^*$-states. In this case their c-work of formation is given by

$$\begin{aligned}
\mathcal{W}_{\mathrm{form}}(\rho^*, N) &= +k_{\mathrm{B}}T \log \gamma_{\mathcal{E}} \\
&= -k_{\mathrm{B}}T \log \left\| \sum_{E\in\mathcal{E}} a_E \right\|_1 \\
&= -k_{\mathrm{B}}T \log \left[\sum_{E\in\mathcal{E}} \sum_{i=1}^{D} \frac{g_{N-1}(E-E_i)}{Z_{\mathrm{S}}^N} e^{-\beta E} \right] \\
&= -k_{\mathrm{B}}T \log \left[\sum_{E\in\mathcal{E}} \frac{1}{Z_{\mathrm{S}}^N} e^{-\beta E} \sum_{i=1}^{D} g_{N-1}(E-E_i) \right] \\
&= -k_{\mathrm{B}}T \log \left[\sum_{E\in\mathcal{E}} \frac{g_N(E)}{Z_{\mathrm{S}}^N} e^{-\beta E} \right],
\end{aligned} \tag{110}$$

where we use that $g_N(E) = \sum_{i=1}^{D} g_{N-1}(E - E_d)$. Let's start with the case where $Np_1, Np_2, \ldots, Np_D \in \mathbb{N}$, then by Lemma 2 we have that $N\langle E\rangle \in \mathcal{E}_N$, where $\langle E\rangle = \sum_{i=1}^{D} p_i E_i$ is the mean energy of a single copy. In such a case, we can bound the sum in Supplementary Equation (110) by a single term in the following way

$$\begin{aligned}
\mathcal{W}_{\mathrm{form}}(\rho^*, N) &= -k_{\mathrm{B}}T \log \left[\sum_{E\in\mathcal{E}} \frac{g_N(E)}{Z_{\mathrm{S}}^N} e^{-\beta E} \right] \\
&\leq -k_{\mathrm{B}}T \log \left[\frac{g_N(N\langle E\rangle)}{Z_{\mathrm{S}}^N} e^{-\beta N\langle E\rangle} \right] \\
&= N\langle E\rangle + Nk_{\mathrm{B}}T \log Z_{\mathrm{S}} - k_{\mathrm{B}}T \log \left[g_N(N\langle E\rangle) \right].
\end{aligned} \tag{111}$$

Notice that the degeneracy $g_N(N\langle E\rangle)$ is grater than or equal to the number of configurations of N copies with Np_1 copies with energy E_1, Np_2 copies with energy E_2, etc. That is, it holds

$$\begin{aligned}
g_N\left(N\langle E\rangle\right) &\geq \binom{N}{Np_1}\binom{N-Np_1}{Np_2}\cdots\binom{N-Np_1-\ldots-Np_{D-1}}{Np_D} \\
&= \frac{N!}{(Np_1)!(Np_2)!\ldots(Np_D)!}.
\end{aligned} \tag{112}$$

Applying the logarithm and using Stirling's approximation we get

$$g_N(N\langle E\rangle) \geq -\sum_{i=1}^{D} Np_i \log p_i + \mathcal{O}(\log N) = NS(\rho^*) + \mathcal{O}(\log N), \tag{113}$$

where $S(\rho^*)$ is the von Neumann entropy of the local state $\rho^* = \sum_{i=1}^{D} p_i \left|E_i\right\rangle\!\left\langle E_i\right|$. From Supplementary Equation (111) we have that

$$\begin{aligned}
\mathcal{W}_{\mathrm{form}}(\rho^*, N) &\leq N\langle E\rangle + Nk_{\mathrm{B}}T \log Z_{\mathrm{S}} - k_{\mathrm{B}}T \log \left[g_N(N\langle E\rangle) \right] \\
&\leq N\langle E\rangle + Nk_{\mathrm{B}}T \log Z_{\mathrm{S}} - k_{\mathrm{B}}TS(\rho^*) + \mathcal{O}(\log N) \\
&= N\left[\langle E\rangle - k_{\mathrm{B}}TS(\rho^*)\right] + Nk_{\mathrm{B}}T \log Z_{\mathrm{S}} + \mathcal{O}(\log N) \\
&= N\left[F(\rho^*) - F(\tau)\right] + \mathcal{O}(\log N).
\end{aligned} \tag{114}$$

Finally, like in the case $D = 2$, using Supplementary Equation (71) we can obtain the lower bound

$$N\Delta F(\rho^*) \leq \mathcal{W}_{\mathrm{form}}(\rho^*, N) \leq N\Delta F(\rho^*) + \mathcal{O}(\log N). \tag{115}$$

If there exists d such that $p_d N \notin \mathbb{N}$, we just replace $\langle E\rangle$ with an approximation of the mean energy $\langle \tilde{E}\rangle$ with $|\langle \tilde{E}\rangle - \langle E\rangle| < D/N$ and the probabilities p_d with $\tilde{p}_d$ with $|\tilde{p}_d - p_d| < 1/N$. These constructions were provided in the previous section when we analyzed the density of reversible states. Thus, when $N \to \infty$ we have $\langle \tilde{E}\rangle \to \langle E\rangle$ and $\tilde{p}_d \to p_d$ and we recover the same results.

The upper bound of Supplementary Equation (114), while it was derived for optimal reversible states, is rather general. It is valid in the large N limit for every multipartite state that: (i) is reversible, (ii) satisfies the partial trace condition, and (iii) it has the mean energy level in its support. In particular, we have derived a family of states σ_d in Supplementary Equation (102) that fulfill these three properties for every local state ρ. Since these states are not optimal, we have that $\mathcal{W}_{\text{form}}(\rho^*, N) \leq W_{\text{form}}(\sigma_d)$ and also $W_{\text{form}}(\sigma_d) \leq N\Delta F(\rho) + \mathcal{O}(\log N)$. Thus, for every state ρ we have that:

$$\Delta F(\rho) \leq \frac{\mathcal{W}_{\text{form}}(\rho, N)}{N} \leq \Delta F(\rho) + \mathcal{O}\left(\frac{\log N}{N}\right). \tag{116}$$

Therefore, the c-work of formation of any state converges to the standard thermodynamic free energy difference in the thermodynamic limit, $N \to \infty$, at a rate $\mathcal{O}\left(\log(N)/N\right)$.

On the other hand, while we cannot obtain the analytical expression of the optimal state in order to obtain the extractable work (since it depends on the specific spectra of each case) we have shown that there exist a family of reversible states that in the thermodynamic limit attain the minimum work cost per particle, recovering standard results from thermodynamics.

Supplementary Note 7. Generalization to different local states

Up to now we have limited ourselves to study the task of creating N copies of the same local state ρ. An obvious generalization of the previous results is to allow each of the N subsystems to have a different local state with an arbitrary Hamiltonian. That is to say, given a set of local states $\{\rho_i\}_{i=1,\ldots,N}$ (of arbitrary different dimensions $\{d_i\}_{i=1,\ldots,N}$ and with arbitrary local Hamiltonian $\{H_i\}_{i=1,\ldots,N}$), we are now interested in creating a global state $\rho^{(N)}$ such that

$$\text{tr}_{-i}\left(\rho^{(N)}\right) = \rho_i, \quad i = 1, \ldots, N. \tag{117}$$

Furthermore, just like before, it is of particular interest to find the state $\rho^{(N)}_{\text{min}}$ that minimizes the work of formation among all the states that satisfy the constraints of Supplementary Equation (117). Now the optimal state will in general not belong to the subset $\mathcal{C}^*$, given that our system is no longer symmetric under permutations. Nonetheless, the optimal solution still is very similar to the previous one.

Corollary 2 (Corollary of Theorem 2). The state $\rho^{(N)}_{\text{min}}$ that satisfies the constraints of Supplementary Equation (117) and minimizes the work of formation has the form

$$\left[\rho^{(N)}_{\text{min}}\right]_i = \gamma\,[\tau]_i\,\mathbb{1}_{\{i\in U\}} + \sum_{j=1}^{M} \gamma s_j\,[\tau]_i\,\mathbb{1}_{\{i=n_j\}}, \quad i \in \{1, 2, \ldots, D_N\}, \tag{118}$$

where $\tau = \tau_1 \otimes \tau_2 \otimes \cdots \otimes \tau_N$ with $\tau_k = e^{-\beta H_k}/Z_k$, $k = 1, \ldots, N$ is the N-partite thermal state; $D_N = \prod_{i=1}^N d_i$; $M \leq \sum_{i=1}^N d_i - N + 1$; $s_1, s_2, \ldots, s_M \in [0, 1]$; $\gamma \in \mathbb{R}_{>0}$; U is a set of indexes where $\left\{\left[\rho^{(N)}_{\text{min}}\right]_i / [\tau]_i\right\}_{i=1,\ldots,D_N}$ realizes its infinity norm; and $n_1, n_2, \ldots, n_M \in \{1, 2, \ldots, D_N\}$.

Proof. This result is immediate from Theorem 2. Indeed we now seek to minimize the infinity norm of a vector q given by

$$q_i = \frac{\left[\rho^{(N)}_{\text{min}}\right]_i}{[\tau]_i} \tag{119}$$

subject to the linear constraints of the partial trace given in Supplementary Equation (117).

Notice now that in the proof of Theorem 2, the details of the matrix A, that codifies the linear constrains on the vector whose infinity norm we are minimizing, is irrelevant in the proof of the general solution Supplementary Equation (78). Therefore, the same solution must also hold for the linear constraints Supplementary Equation (117) in the case where each local system is different.

Similarly to the previous cases, again we will have particular cases where the optimal state is reversible. This will happen whenever the local states are such that in the optimal solution we have that $s_1 = s_2 = \cdots = s_M = 0$ or $s_1 = s_2 = \cdots = s_M = 1$.

A. Thermodynamic limit

In this last section we are going to prove a generalization of our results in the thermodynamic limit.

Theorem 3 (Theorem 3 in the main text). Let $(p^{(1)}, E^{(1)}), (p^{(2)}, E^{(2)}), \dots, (p^{(N)}, E^{(N)}) \in \mathbb{R}^D \otimes \mathbb{R}^D$ be an i.i.d sample with arbitrary distribution $\mathcal{D}$ such that the maximum level of energy is upper bounded, and $\mathcal{W}_N$ the c-work of formation of a system with block-diagonal reduced states ρ_i defined by the probability vector $p^{(i)}$ and Hamiltonian with energies $E^{(i)}$. Then,

$$\frac{\mathcal{W}_N}{N} \xrightarrow{N\to\infty} \langle \Delta F \rangle_{\mathcal{D}}\,, \tag{120}$$

where the mean in ΔF is with respect to $\mathcal{D}$ and the convergence is almost surely.

The almost surely convergence in Theorem 3 means that given $\epsilon > 0$, there exists $N_0 < \infty$ such that

$$\left| \frac{\mathcal{W}_N}{N} - \langle \Delta F \rangle_{\mathcal{D}} \right| < \epsilon \qquad \text{for all } N > N_0. \tag{121}$$

Notice that N_0 is also a random variable that takes a finite value.

Proof. First, we are going to analyze the case where the support of $\mathcal{D}$ is discrete and finite. The extension to absolutely continuous, singular continuous and discrete distribution with numerable support follow from the first case. Notice that these cases allows us to say that $\mathcal{D}$ is an arbitrary distribution (see Lebesgue's decomposition theorem) and then we are not making any extra assumption on $\mathcal{D}$ except that $\langle \Delta F \rangle_{\mathcal{D}}$ is well defined.

Let's consider first the case where $\mathcal{D}$ consists of a discrete distribution with support in a finite set

$$\{(p_1^\star, E_1^\star), (p_2^\star, E_2^\star), \dots, (p_K^\star, E_K^\star)\} \subset \mathbb{R}^D \times \mathbb{R}^D, \tag{122}$$

and denote the probability of taking each one of these values as

$$\mathbb{P}\left(p_i = p_j^\star, E_i = E_j^\star\right) = r_j, \quad \sum_{j=1}^K r_j = 1, \quad r_j > 0. \tag{123}$$

If $n_j := \sum_{i=1}^N \mathbb{I}_{\{p_i = p_j^\star, E_i = E_j^\star\}}$ is the number of subsystems with eigenvalues $p_j^\star$ and respective energies $E_j^\star$, then $(n_1, \dots, n_K)$ is distributed as a multinomial distribution with N numbers of trials and parameter $(r_1, \dots, r_K)$. Given $\epsilon_2 > 0$, let us consider the event

$$\Omega_{\epsilon_2,N} = \Big\{ (1-\epsilon_2) r_j N \leq n_j \leq (1+\epsilon_2) r_j N \text{ for all } j = 1, \dots, K \Big\}. \tag{124}$$

Using the Hoeffding inequality we can prove that the probability of the complement of $\Omega_{\epsilon_2,N}$ is bounded as

$$\begin{aligned}
\mathbb{P}\left(\Omega^c_{\epsilon_2,N}\right) &= \mathbb{P}\left(\bigcup_{j=1}^K \Big\{ |n_j - N r_j| > \epsilon_2 r_j N \Big\} \right) \\
&\leq \sum_{j=1}^K \mathbb{P}\Big(|n_j - N r_j| > \epsilon_2 r_j N \Big) \\
&\leq \sum_{j=1}^K 2 e^{-2\epsilon_2^2 r_j^2 N},
\end{aligned} \tag{125}$$

that is, except for a probability exponentially small in N the system satisfies $\Omega_{\epsilon_2,N}$. On the other hand, on $\Omega_{\epsilon_2,N}$ for $\rho_j^\star = \sum_{d=1}^D (p_j^\star)_d \left|(E_j^\star)_d\middle\rangle\!\middle\langle(E_j^\star)_d\right|$ it holds that

$$\begin{aligned}
\mathcal{W}_N &\leq \sum_{j=1}^K \mathcal{W}_{\text{form}}(n_j, \rho_j^\star) \\
&\leq \sum_{j=1}^K (1+\epsilon_2) r_j N \Delta F(\rho_j^\star) + \mathcal{O}(\log n_j) \\
&= (1+\epsilon_2) N \langle \Delta F \rangle_{\mathcal{D}} + \mathcal{O}(K \log N),
\end{aligned} \tag{126}$$

where we use the result from the thermodynamics limit for copies. Notice that the correlations that are needed in order to bound $\mathcal{W}_N$ just include the identical subsystems. In the same way from the subadditivity of the von Neumann entropy it follows that $(1-\epsilon_2)N\langle\Delta F\rangle_{\mathcal{D}} < \mathcal{W}_N$ on $\Omega_{\epsilon_2,N}$. Let's consider ϵ_2 such that $2\epsilon_2\langle\Delta F\rangle_{\mathcal{D}} < \epsilon$. On the other hand, since the $\mathcal{O}(K\log N)$ is just an additive constant, there exists $N_0 = N_0(\epsilon)$ such that it is upper bounded by $\epsilon N/2$ for all $N > N_0$. Finally,

$$\left|\frac{\mathcal{W}_N}{N} - \langle\Delta F\rangle_{\mathcal{D}}\right| < \epsilon \qquad \text{for all } N > N_0 \text{ on } \Omega_{\epsilon_2,N}. \tag{127}$$

Since $\sum_{N=1}^{\infty}\mathbb{P}(\Omega^c_{\epsilon_2,N}) < \infty$, from the Borel-Cantelli lemma it follows that $\mathcal{W}_N/N$ converges almost surely to the mean value of the free energy difference.

Now we are going to generalize the proof for a more generic class of distributions $\mathcal{D}$. Let $\{p^{(i)}, E^{(i)}\}_{i=1,2,\ldots,N}$ be distributed according to a general distribution $\mathcal{D}$ on $\mathbb{R}^{2D}_{\geq 0}$. The distribution in energy is upper bounded by a maximum energy $E_{\max}$. From the subadditivity of the von Neumann entropy is clear that

$$\langle\Delta F\rangle_{\mathcal{D}} \leq \lim_{N\to\infty}\frac{\mathcal{W}_N}{N}. \tag{128}$$

Let's prove the other inequality. First, notice that if we consider a disjoint partition $V_1, V_2, \ldots V_R$ of the support of the distribution $\mathcal{D}$, then it is enough to prove that

$$\frac{\mathcal{W}_N^{V_j}}{N} \xrightarrow{N\to\infty} \mathbb{E}[\Delta F|V_j]\mathbb{P}(V_j) \quad \text{almost surely for all } j = 1,2,\ldots,R, \tag{129}$$

where $\mathcal{W}_N^{V}$ represents the correlated work of formation of those subsystems with probabilities and energies on V; $\mathbb{E}[\Delta F|V]$ is the expectation value of the difference of free energy conditional to V; and $\mathbb{P}(V)$ is the probability under $\mathcal{D}$ of V. This follows from the fact that

$$\frac{\mathcal{W}_N}{N} - \langle\Delta F\rangle_{\mathcal{D}} \leq \sum_{j=1}^{R}\frac{\mathcal{W}_N^{V_j}}{N} - \mathbb{E}[\Delta F|V_j]\mathbb{P}(V_j). \tag{130}$$

Let us consider the function ψ from $\{0,1\}^D$ to the sets of $\mathbb{R}^D$ defined as $\psi(a_1,\ldots,a_D) = \{p \in \mathbb{R}^D : p_i = 0 \text{ if } a_i = 0 \text{ and } p_i > 0 \text{ if } a_i = 1\}$. Notice that the family of sets $\mathcal{V}$ with elements $\psi(a_1,\ldots,a_D)\times[0,E_{\max}]$ for $a_i = 0,1$ and $a_1+\ldots+a_D \geq 1$ defines a disjoint partition of the support of $\mathcal{D}$. In particular, if $\mathcal{D}$ is an absolute continuous distribution, the only set of this family with non-zero probability is $V_0 := \{(p,E) \in \mathbb{R}^{2D} : \min_i p_i > 0\}$. For the sake of explanation, let us consider this case and then we generalize our results to the case where more elements with non-zero probability are present on $\mathcal{V}$.

Since $\mathbb{P}(V_0) = 1$, we can omit the conditionals V_0 in each one of the previous expressions. Given $n \in \mathbb{N}$, we define

$$K_n = \left\{(p,E) \in \mathbb{R}^{2D} : \min_i p_i \geq 1/n\right\}. \tag{131}$$

Then we have that $\mathbb{P}(K_n) \to 1$ as $n \to \infty$. Given $\epsilon_3 > 0$, let n be such that $\mathbb{P}(K_n^c) < \epsilon_3$. Now, we can define $\Delta F_{\max} = \max_\rho \Delta F(\rho)$ where the maximum is taken respect all the possible states ρ with energy distribution bounded by $E_{\max}$. Since

$$\langle\Delta F\rangle_{\mathcal{D}} = \mathbb{E}\left[\Delta F|K_n\right]\mathbb{P}(K_n) + \mathbb{E}\left[\Delta F|K_n^c\right]\mathbb{P}(K_n^c), \tag{132}$$

it follows that

$$\langle\Delta F\rangle_{\mathcal{D}} - \mathbb{E}\left[\Delta F|K_n\right]\mathbb{P}(K_n) \leq \epsilon_3\Delta F_{\max}. \tag{133}$$

On the other hand, $\mathcal{W}_N \leq \mathcal{W}_N^{\mathbb{K}_n} + \mathcal{W}_N^{\mathbb{K}_n^c}$, where $\mathcal{W}_N^{\mathbb{K}_n}$ is the correlated work of formation of the systems with probabilities and energies in $\mathbb{K}_n = \{(p^{(i)},E^{(i)}) : (p^{(i)},E^{(i)}) \in K_n\}$. Notice that in the set $\Omega_{\epsilon_3} = \{\#\mathbb{K}_n^c < 2\epsilon_3 N\}$ it holds $\mathcal{W}_N^{\mathbb{K}_n^c} \leq 2\epsilon_3 N W_{\text{form}}^{\max}$, where $W_{\text{form}}^{\max}$ is the maximum work of formation of all the states ρ with energy distribution bounded by $E_{\max}$. Given $\epsilon > 0$, if we choose $\epsilon_3 = \epsilon/2(2W_{\text{form}}^{\max} + \Delta F_{\max})$, then

$$\frac{\mathcal{W}_N}{N} - \langle\Delta F\rangle_{\mathcal{D}} < \frac{\mathcal{W}_N^{\mathbb{K}_n}}{N} - \mathbb{E}\left[\Delta F|K_n\right]\mathbb{P}(K_n) + \frac{\epsilon}{2} \quad \text{on } \Omega_{\epsilon_3}. \tag{134}$$

On the other hand,

$$\frac{\mathcal{W}_N^{\mathbb{K}_n}}{N} - \mathbb{E}\left[\Delta F|K_n\right]\mathbb{P}(K_n) = \frac{\mathcal{W}_N^{\mathbb{K}_n}}{\#\mathbb{K}_n}\frac{\#\mathbb{K}_n}{N} - \mathbb{E}\left[\Delta F|K_n\right]\mathbb{P}(K_n)$$
$$< \left(\frac{\mathcal{W}_N^{\mathbb{K}_n}}{\#\mathbb{K}_n} - \mathbb{E}\left[\Delta F|K_n\right]\right) + \left(\mathbb{E}\left[\Delta F|K_n\right]\left(\frac{\#\mathbb{K}_n}{N} - \mathbb{P}(K_n)\right)\right). \tag{135}$$

Since $\#\mathbb{K}_n/N \to \mathbb{P}(K_n)$ almost surely, there exists $N_2 < \infty$ such that the second term in Supplementary Equation (135) is smaller than $\epsilon/4$ for all $N > N_2$. On the contrary, the first term corresponds to the thermodynamic limit for the subset of systems in $\mathbb{K}_n$. In order to use the result for discrete and finite distributions we need the following lemma. The proof of the lemma is given at the end of the supplementary material.

Lemma 3 (Continuity of Thermo-majorization). Given $w > 0$, and a state $\rho(p,E) := \sum_{i=1}^{D} p_i \left|E_i\right\rangle\!\left\langle E_i\right|$, with $(p,E) \in \mathbb{R}^{2D}$, there exists $\delta = \delta(w,p,E)$ such that for any other $(\tilde{p},\tilde{E}) \in \mathbb{R}^{2D}$

- if $\|(p,E) - (\tilde{p},\tilde{E})\| < \delta$ then the transformation
$$\rho(p,E) \otimes |w\rangle\!\langle w| \to \rho(\tilde{p},\tilde{E}) \otimes |0\rangle\!\langle 0| \tag{136}$$
can be performed with thermal operations;
- if $\|(p,E) - (\tilde{p},\tilde{E})\| < \delta$ and $p_i = 0$ implies $\tilde{p}_i = 0$, then the transformation
$$\rho(\tilde{p},\tilde{E}) \otimes |w\rangle\!\langle w| \to \rho(p,E) \otimes |0\rangle\!\langle 0| \tag{137}$$
can be performed with thermal operations.

In the previous lemma, the distance measure between probabilities and energies could be chosen as any of the vector norms in $\mathbb{R}^{2D}$, for example, the infinity norm given by $\|(p,E) - (\tilde{p},\tilde{E})\|_\infty := \max\{\|p - \tilde{p}\|_\infty, \|E - \tilde{E}\|_\infty\}$. On the other hand, notice that the condition $\tilde{p}_i = 0$ if $p_i = 0$ is equivalent to the condition of $\mathrm{supp}(\rho(\tilde{p},\tilde{E})) \subseteq \mathrm{supp}(\rho(p,E))$ when $E = \tilde{E}$.

With the previous considerations, we need to prove the theorem for a distribution supported on K_n with $n = n(\epsilon)$ chosen as before. Let's consider $w = \epsilon/16$ and for each pair $(p,E) \in K_n$ we select $\delta_2(p,E)$ as in Lemma 3. On the other hand, $\Delta F(\rho)$ is a continuous function of (p,E). Since K_n is a compact set, $\Delta F(\rho)$ is also absolutely continuous and there exists δ_0 such that if $\|(p,E) - (\tilde{p},\tilde{E})\| < \delta_0$ then $|\Delta F(\rho) - \Delta F(\tilde{\rho})| < \epsilon/16$. Let's define $\delta(p,E) = \min\{\delta_0, \delta_2(p,E)\}$. Then we can cover K_n with the family of open balls with center in (p,E) and radius $\delta(p,E)$ as

$$K_n \subset \bigcup_{(p,E)\in K_n} B((p,E),\delta(p,E)). \tag{138}$$

Since K_n is a compact set on $\mathbb{R}^{2D}$, we can select a finite family $\{(p_j^*,E_j^*)\}_{j=1,2,\ldots,K}$ such that

$$K_n \subset \bigcup_{j=1}^{K} B\left((p_j^*,E_j^*),\delta(p_j^*,E_j^*)\right). \tag{139}$$

Let us define the family of sets

$$V_1 = B\left((p_1^*,E_1^*),\delta(p_j^*,E_j^*)\right) \cap K_n, \tag{140}$$
$$V_j = B\left((p_j^*,E_j^*),\delta(p_j^*,E_j^*)\right) \cap K_n \setminus \cup_{l=1}^{j-1} B\left((p_l^*,E_l^*),\delta(p_l^*,E_l^*)\right), \quad j = 2,\ldots,K, \tag{141}$$

and their respective probabilities $r_j = \mathbb{P}(V_j)$. Notice that $V_1, V_2, \ldots V_K$ is a partition of K_n. If $\tilde{\mathcal{D}}$ denotes the discrete and finite distribution with support in $\{(p_j^*,E_j^*)\}_{j=1,2,\ldots,K}$ and pointwise probabilities given by $r_1, r_2, \ldots, r_K$, then

$$\frac{\mathcal{W}_N^{\mathbb{K}_n}(\mathcal{D})}{\#\mathbb{K}_n} - \mathbb{E}_{\mathcal{D}}\left[\Delta F|K_n\right] < \left(\frac{\mathcal{W}_N^{\mathbb{K}_n}(\tilde{\mathcal{D}})}{\#\mathbb{K}_n} - \mathbb{E}_{\tilde{\mathcal{D}}}\left[\Delta F|K_n\right]\right) + \left(\frac{\mathcal{W}_N^{\mathbb{K}_n}(\mathcal{D})}{\#\mathbb{K}_n} - \frac{\mathcal{W}_N^{\mathbb{K}_n}(\tilde{\mathcal{D}})}{\#\mathbb{K}_n}\right) + \left(\mathbb{E}_{\tilde{\mathcal{D}}}\left[\Delta F|K_n\right] - \mathbb{E}_{D}\left[\Delta F|K_n\right]\right)$$
$$< \left|\frac{\mathcal{W}_N^{\mathbb{K}_n}(\tilde{\mathcal{D}})}{\#\mathbb{K}_n} - \mathbb{E}_{\tilde{\mathcal{D}}}\left[\Delta F|K_n\right]\right| + w + \frac{\epsilon}{16}. \tag{142}$$

In the last equation we have used that $\mathcal{W}_N^{\mathbb{K}_n}(\mathcal{D})-\mathcal{W}_N^{\mathbb{K}_n}(\tilde{\mathcal{D}}) < \#\mathbb{K}_n w$. In order to arrive at this bound we use Lemma 3, which tells us that we can transform a state defined by (p_j^*, E_j^*) into any other state in $B((p_j^*, E_j^*), \delta(p_j^*, E_j^*))$ using an amount of work w. Now, we can consider the following two $\#\mathbb{K}_n$-partite optimal states, the first one $\rho_{\tilde{\mathcal{D}}}^{(\#\mathbb{K}_n)}$ is defined by the discrete distribution $\tilde{\mathcal{D}}$ with support in $\{(p_j^*, E_j^*)\}_{j=1,2,\ldots,K}$ and work of formation $\mathcal{W}_N^{\mathbb{K}_n}(\tilde{\mathcal{D}})$; the other $\rho_{\mathcal{D}}^{(\#\mathbb{K}_n)}$ is defined by the distribution $\mathcal{D}$ with work of formation $\mathcal{W}_N^{\mathbb{K}_n}(\mathcal{D})$. Let us also assume that $\mathcal{W}_N^{\mathbb{K}_n}(\mathcal{D}) \geq \mathcal{W}_N^{\mathbb{K}_n}(\tilde{\mathcal{D}})$ (if this is not the case we can bound the above term with $w = 0$). Then, notice that with an amount of work $(\#\mathbb{K}_n\, w)$ we can transform $\rho_{\tilde{\mathcal{D}}}^{(\#\mathbb{K}_n)} \to \tilde{\rho}_{\mathcal{D}}^{(\#\mathbb{K}_n)}$, this is done by applying locally to each subsystem the thermal operation that transform with work w the state with (p_j^*, E_j^*) to the corresponding state (p, E) defined by $\mathcal{D}$. This transformation guarantees that the state $\tilde{\rho}_{\mathcal{D}}^{(\#\mathbb{K}_n)}$ is locally equivalent to the optimal state $\rho_{\mathcal{D}}^{(\#\mathbb{K}_n)}$, and allows us to bound $\mathcal{W}_N^{\mathbb{K}_n}(\mathcal{D})$. Thus, $\mathcal{W}_N^{\mathbb{K}_n}(\tilde{\mathcal{D}}) + (\#\mathbb{K}_n\, w) \geq \tilde{\mathcal{W}}_N^{\mathbb{K}_n}(\tilde{\mathcal{D}}) \geq \mathcal{W}_N^{\mathbb{K}_n}(\mathcal{D})$, with $\tilde{\mathcal{W}}_N^{\mathbb{K}_n}(\mathcal{D})$ the work of formation of $\tilde{\rho}_{\mathcal{D}}^{(\#\mathbb{K}_n)}$.

Finally, all that remains is to bound the first term which corresponds to the thermodynamic limit for the discrete distribution. By the previous results, this can be done by taking N_1 such that

$$\left| \frac{\mathcal{W}_N^{\mathbb{K}_n}(\tilde{\mathcal{D}})}{\#\mathbb{K}_n} - \mathbb{E}_{\tilde{\mathcal{D}}}\left[\Delta F | K_n\right] \right| < \frac{\epsilon}{8} \quad \text{for all } N > N_1. \tag{143}$$

Notice that the main ingredient of the proof was that we can restrict the analysis to a compact subset with elements that have the same support (in order to use Lemma 3). The same analysis can been performed in each set in $V \in \mathcal{V}$ with the topology of the open sets restricted to V.

Proof (Proof of Lemma 3). Let us consider $E_1 \leq E_2, ..., \leq E_D$ and a β-order $\pi(1), \pi(2), \ldots, \pi(D)$ of $1, 2, \ldots, D$ such that

$$p_{\pi(1)} e^{\beta E_{\pi(1)}} \geq p_{\pi(2)} e^{\beta E_{\pi(2)}} \geq \ldots \geq p_{\pi(D)} e^{\beta E_{\pi(D)}}. \tag{144}$$

Given another $(\tilde{p}, \tilde{E})$, we can choose $\delta_1 > 0$ such that if $\|(p, E) - (\tilde{p}, \tilde{E})\| < \delta_1$ then the same ordering holds, that is,

$$\tilde{p}_{\pi(1)} e^{\beta \tilde{E}_{\pi(1)}} \geq \tilde{p}_{\pi(2)} e^{\beta \tilde{E}_{\pi(2)}} \geq \ldots \geq \tilde{p}_{\pi(D)} e^{\beta \tilde{E}_{\pi(D)}}. \tag{145}$$

If all the $\geq$ are actually $>$, then this follows directly selecting δ_1 small enough. On the other hand, if there exists i such that $p_{\pi(i)} e^{\beta E_{\pi(i)}} = p_{\pi(i+1)} e^{\beta E_{\pi(i+1)}}$, we can change the ordering π for another $\tilde{\pi}$ with $\tilde{\pi}(i) = \pi(i+1)$ and $\tilde{\pi}(i+1) = \pi(i)$ in order to ensure the ordering. For simplicity, in the following analysis we are going to use i instead of $\pi(i)$.

The necessary and sufficient condition to ensure that the transformation $\rho(p, E) \otimes |w\rangle\langle w| \to \rho(\tilde{p}, \tilde{E}) \otimes |0\rangle\langle 0|$ is possible via thermal operations is thermo-majorization [1], which means that the polygonal defined by the vertices on $(x_0, y_0) = (0, 0), (x_1, y_1), \ldots, (x_D, y_D)$, with $x_i = e^{-\beta w}(e^{-\beta E_1} + \ldots + e^{-\beta E_i})$ and $y_i = p_1 + \ldots + p_i$, lies completely above the polygonal with vertices on $(\tilde{x}_0, \tilde{y}_0) = (0, 0), (\tilde{x}_1, \tilde{y}_1), \ldots, (\tilde{x}_D, \tilde{y}_D)$, with $\tilde{x}_i = e^{-\beta \tilde{E}_1} + \ldots + e^{-\beta \tilde{E}_i}$ and $y_i = \tilde{p}_1 + \ldots + \tilde{p}_i$. If we choose $\|E - \tilde{E}\|_\infty < w$, then it holds

$$e^{-\beta w}\left(e^{-\beta E_1} + \ldots + e^{-\beta E_i}\right) < e^{-\beta \tilde{E}_1} + \ldots + e^{-\beta \tilde{E}_i}, \tag{146}$$

for all $i = 1, 2, \ldots, D$, which is equivalent to $x_i < \tilde{x}_i$. Equivalently, if w is such that

$$w < \delta_2 := \frac{1}{2} k_{\mathrm{B}} T \max_{i=1,\ldots,D} \log\left(1 + \frac{e^{-\beta E_i}}{e^{-\beta E_1} + \ldots + e^{-\beta E_D}}\right), \tag{147}$$

then $\tilde{x}_i < x_{i+1}$. Notice that the condition in Supplementary Equation (147) is not restrictive.

With the previous considerations, if we choose $\delta < \min\{\delta_1, \delta_2, w\}$ then the thermo-majorization condition will be satisfied if each $(\tilde{x}_i, \tilde{y}_i)$ lies below the line that connects (x_i, y_i) with (x_{i+1}, y_{i+1}), that is

$$\tilde{y}_i \leq y_i + \frac{y_{i+1} - y_i}{x_{i+1} - x_i}(\tilde{x}_i - x_i). \tag{148}$$

For those i such this $p_{i+1} \neq 0$, that is equivalent to

$$\frac{e^{-\beta E_{i+1}}}{p_{i+1}} \sum_{j=1}^{i} (\tilde{p}_j - p_j) \leq e^{\beta w}\left(e^{-\beta \tilde{E}_1} + \ldots + e^{-\beta \tilde{E}_i}\right) - \left(e^{-\beta E_1} + \ldots + e^{-\beta E_i}\right). \tag{149}$$

The condition of Supplementary Equation (149) will be simultaneously satisfied for all i with $p_i \neq 0$ if

$$e^{\beta\|E-\tilde{E}\|_\infty}\left(\frac{e^{\beta E_1}}{\min_{i:p_i\neq 0} p_i e^{\beta E_i}}\|p-\tilde{p}\|_1+1\right)\leq e^{\beta w}. \tag{150}$$

Then, we can choose $\delta=\min\{\delta_1,\delta_2,\delta_3,w\}$ with δ_3 such that if $\|p-\tilde{p}\|_1<\sqrt{D}\|p-\tilde{p}\|_\infty<\delta_3$ and $\|E-\tilde{E}\|_\infty<\delta_3$ then Supplementary Equation (150) holds. When the probability vectors are equal, it is just the maximum energy difference that bounds the work w. On the other hand, if $p_{i+1}=0$ then Supplementary Equation (148) is trivially satisfied since $y_i=1$.

Let's now study the transformation $\rho(\tilde{p},\tilde{E})\otimes|w\rangle\langle w|\to\rho(p,E)\otimes|0\rangle\langle 0|$. Just like before, we can choose $\delta_1>0$ such that if $\|(p,E)-(\tilde{p},\tilde{E})\|_1<\delta_1$, the thermo-majorization order does not change. The first polygonal now is defined as $(x_0,y_0)=(0,0),(x_1,y_1),\dots,(x_D,y_D)$ with $x_i=e^{-\beta w}(e^{-\beta\tilde{E}_1}+\ldots+e^{-\beta\tilde{E}_i})$ and $y_i=\tilde{p}_1+\ldots+\tilde{p}_i$; and the other (which has to lie completely below the first one) by $(\tilde{x}_0,\tilde{y}_0)=(0,0),(\tilde{x}_1,\tilde{y}_1),\dots,(\tilde{x}_D,\tilde{y}_D)$ with $\tilde{x}_i=e^{-\beta E_1}+\ldots+e^{-\beta E_i}$ and $\tilde{y}_i=p_1+\ldots+p_i$. Proceeding like before, there exists $\delta_2>0$ such that if $\|E-\tilde{E}\|_\infty<\delta_2$ then $\tilde{x}_{i-1}<x_i<\tilde{x}_i$ for all $i=1,2,\dots,D$.

If $\tilde{x}_{i-1}<x_i<\tilde{x}_i$, then a sufficient condition is that (x_i,y_i) lies above the line that connects $(\tilde{x}_i,\tilde{y}_i)$ with $(\tilde{x}_{i+1},\tilde{y}_{i+1})$, that is,

$$y_i\geq\tilde{y}_i-\frac{\tilde{y}_{i+1}-\tilde{y}_i}{\tilde{x}_{i+1}-\tilde{x}_i}(\tilde{x}_i-x_i). \tag{151}$$

If $p_{i+1}\neq 0$, this is equivalent to

$$\frac{e^{-\beta E_{i+1}}}{p_{i+1}}\sum_{j=1}^{i}(p_j-\tilde{p}_j)\leq\left(e^{-\beta E_1}+\ldots+e^{-\beta E_i}\right)-e^{\beta w}\left(e^{-\beta\tilde{E}_1}+\ldots+e^{-\beta\tilde{E}_i}\right). \tag{152}$$

A sufficient condition to ensure that Supplementary Equation (152) holds is Supplementary Equation (150). On the other hand, if $p_{i+1}=0$ then we need $y_i=\tilde{p}_1+\ldots+\tilde{p}_i\geq\tilde{y}_i=1$, which is satisfied if and only if $\tilde{p}_j=0$ for all $j>i$. This is true by hypothesis that the support of $\tilde{p}$ is contained in the support of p and the fact that $p_{i+1}=0$ implies that $p_j=0$ for all $j\geq i+1$, since p_i is β-ordered.

Supplementary References

[1] M. Horodecki and J. Oppenheim, Nature Communications **4**, 2059 (2013).

[2] F. Brandao, M. Horodecki, N. Ng, J. Oppenheim, and S. Wehner, Proceedings of the National Academy of Sciences **112**, 3275 (2015).

[3] H.-K. Hwang, Studies in Applied Mathematics **99**, 393 (1997).
